# Distant Brain Failure and Subsequent Survival After Gamma Knife Radiosurgery for Breast Cancer Brain Metastases: Prognostic Value of Pretreatment Inflammatory Markers

**DOI:** 10.3390/jcm15218701

**Published:** 2026-11-07

**Authors:** Sijun Diao, Fujun Liu, Zhongyu Li, Shihong Zhu, Xiyue Lu, Huiyang Luo, Jing Chen

**Affiliations:** 1 Department of Neurosurgery, West China Hospital, West China Medical School, Sichuan University, Chengdu 610041, China; diaosijun4016@163.com (S.D.); lzainleey@163.com (Z.L.); austin_zhushihong@163.com (S.Z.); 2 State Key Laboratory of Biotherapy and Cancer Center, West China Hospital, Sichuan University, Chengdu 610041, China; fjliu2017@163.com; 3 Department of Anesthesiology, West China Hospital, West China Medical School, Sichuan University, Chengdu 610041, China; 15680613113@163.com (X.L.); luohuiyang0216@outlook.com (H.L.)

**Keywords:** breast cancer, brain metastases, Gamma Knife radiosurgery, distant brain failure, neutrophil-to-lymphocyte ratio, competing risks, multistate analysis

## Abstract

**Background/Objectives:** Whether pretreatment inflammatory markers inform both the risk of distant brain failure (DBF) and survival after its occurrence remains unclear in breast cancer brain metastases. We evaluated these associations across the clinical course following Gamma Knife radiosurgery (GKRS). **Methods:** This retrospective cohort included 91 women undergoing initial GKRS. Four blood-derived inflammatory markers were analyzed separately as standardized log_2_-transformed variables. Fine–Gray models evaluated first DBF with death as a competing event. Multistate and transition-specific Cox analyses distinguished mortality before and after DBF. Marker comparisons underwent Benjamini–Hochberg false-discovery-rate correction. **Results:** DBF developed in 57 patients, of whom 36 subsequently died; 16 patients died before DBF. Cumulative DBF incidence reached 41.8% at 12 months and 61.0% at 24 months. A higher pretreatment neutrophil-to-lymphocyte ratio (NLR) and platelet-to-lymphocyte ratio were associated with first DBF (adjusted subdistribution hazard ratios per standard-deviation increase: 1.52, 95% confidence interval [CI] 1.09–2.10; and 1.38, 95% CI 1.04–1.83, respectively; both q = 0.047). Median survival after DBF was 10.5 months. NLR measured before initial GKRS remained associated with post-DBF mortality after adjustment for age, performance status at DBF, and extracranial disease progression at DBF (hazard ratio, 2.29; 95% CI, 1.45–3.61; q = 0.003). With a time-varying performance-status coefficient, the NLR hazard ratio was 2.75 (95% CI, 1.68–4.51; nominal *p* < 0.001). No inflammatory marker was significantly associated with mortality before DBF after adjustment for multiple testing. **Conclusions:** Pretreatment NLR was associated with both first DBF and subsequent mortality, whereas PLR was associated with first DBF. The predominantly HER2-positive cohort (68.1%) limits generalizability. Further validation is needed to establish the predictive and clinical value of these associations.

## 1. Introduction

Brain metastases impose a substantial burden in breast cancer, although their incidence varies considerably by disease stage and molecular subtype. In a population-based study, Martin et al. found brain metastases at diagnosis in 0.41% of all patients with breast cancer and 7.56% of those presenting with metastatic disease [1]. The burden increases over the subsequent disease course. A meta-analysis of 25 studies reported pooled cumulative incidences of 31%, 32%, and 15% in HER2-positive, triple-negative, and hormone receptor-positive/HER2-negative metastatic breast cancer, respectively, over median follow-up periods of approximately 31–33 months [2]. Neurological symptom burden is associated with poorer survival, while discordance in hormone receptor or HER2 expression between primary tumors and brain metastases further complicates disease assessment and treatment selection [3,4]. Prognosis also depends on extracranial disease control, highlighting the need to consider intracranial and systemic disease together [5].

Management of breast cancer brain metastases (BCBM) combines surgery, radiotherapy, and subtype-directed systemic treatment according to neurological symptoms, intracranial disease burden, performance status, and available treatment options [6]. Stereotactic radiosurgery (SRS), including Gamma Knife radiosurgery (GKRS), delivers focal treatment while limiting irradiation of normal brain tissue. In a randomized trial involving patients with one to three brain metastases from different primary cancers, SRS alone resulted in less cognitive deterioration than SRS combined with whole-brain radiotherapy (WBRT), although intracranial failure was more frequent [7]. Favorable local control has also been reported in breast cancer-specific cohorts: an international study of 195 patients with HER2-positive BCBM reported a 12-month local control rate of 94% after SRS [8]. Meanwhile, systemic treatments with intracranial activity have expanded. The updated HER2CLIMB analysis demonstrated improved survival and a reduced risk of new brain lesions with tucatinib, trastuzumab, and capecitabine, while DESTINY-Breast12 showed substantial intracranial activity of trastuzumab deruxtecan in patients with stable or active brain metastases [9,10]. These advances have changed clinical practice, although improvements in outcomes have not been uniform across breast cancer subtypes [11].

Even when existing lesions are effectively treated, new metastases may develop elsewhere in the brain, a pattern of progression termed distant brain failure (DBF). In a contemporary cohort of patients with HER2-positive BCBM, Turna et al. reported local control rates of 98% and 80% at 1 and 2 years, respectively, whereas the corresponding distant brain metastasis-free survival rates were 91% and 63% [12]. These findings underscore the need to assess ongoing intracranial disease beyond the initially treated sites. Established tools, such as the updated Breast Graded Prognostic Assessment, combine clinical and molecular features to estimate survival but were not developed specifically to assess DBF risk or survival after DBF [13]. Biomarker studies have begun to address intracranial outcomes. For example, de Castro et al. prospectively evaluated the relationship between circulating tumor cells and brain disease control after focal radiotherapy for BCBM [14]. Readily available blood markers may offer additional information about the risk of distinct adverse outcomes after GKRS.

Inflammatory indices derived from routine blood counts are practical candidates because they are inexpensive and widely available. A systematic review and meta-analysis associated a higher neutrophil-to-lymphocyte ratio (NLR) with poorer survival in breast cancer, providing a rationale for its evaluation in more specific clinical settings [15]. In a mixed-primary cohort of 195 patients treated with GKRS, Zhang et al. found that higher pretreatment NLR was independently associated with DBF, local failure, and overall survival [16]. Subsequent evidence, however, suggests that its prognostic relevance may depend on the treatment setting and endpoint. Among 185 patients receiving SRS with concurrent immune checkpoint inhibitors, Yomo et al. associated higher NLR with shorter overall and intracranial progression-free survival, but found no significant differences in the cumulative incidence of individual intracranial failure events; only two patients in that cohort had breast cancer [17]. Other indices have also been investigated, including the lymphocyte-to-monocyte ratio (LMR) in patients receiving fractionated stereotactic radiotherapy for brain metastases from non-small-cell lung cancer and the systemic immune-inflammation index (SII) in a mixed-primary SRS cohort [18,19]. These studies support further investigation of inflammatory markers, but their relationship with the intracranial disease course in breast cancer remains incompletely defined.

Several questions therefore remain. Differences in primary cancer, systemic treatment exposure, marker thresholds, and endpoint definitions limit the direct applicability of previous findings to patients with BCBM undergoing GKRS. An association with overall survival or a composite progression endpoint does not necessarily imply an association with first DBF. Death before DBF precludes its subsequent occurrence and must therefore be considered as a competing event when estimating cumulative DBF incidence [20]. Patients who develop DBF enter a different phase of disease, during which functional status at progression and subsequent treatment may influence survival. Whether pretreatment inflammatory markers show similar associations with first DBF, death before DBF, and death after DBF remains unclear.

We therefore conducted a retrospective cohort study of patients with BCBM undergoing their first GKRS to examine the associations of pretreatment NLR, platelet-to-lymphocyte ratio (PLR), SII, and LMR with the subsequent disease course. We estimated the cumulative incidence of first DBF in the presence of competing mortality and separately assessed factors associated with transitions from survival without DBF to DBF or death, and from DBF to death. We also explored the relationships of clinical factors at DBF and subsequent treatment with post-DBF survival. Through this approach, we sought to clarify how readily available inflammatory markers relate to distinct stages of disease after GKRS.

## 2. Materials and Methods

### 2.1. Study Design and Patient Selection

This single-center retrospective cohort study included patients with breast cancer brain metastases (BCBM) who underwent first Gamma Knife radiosurgery (GKRS) at West China Hospital, Sichuan University, between 1 July 2021 and 1 July 2025. A total of 133 patients identified from the institutional screening register were assessed for eligibility. Baseline was defined as the date of first GKRS, and the follow-up cutoff date was 1 July 2026.

Patients were eligible if they had pathologically confirmed breast cancer, radiologically confirmed brain metastases, pretreatment blood counts sufficient to calculate the inflammatory indices, and clinical records allowing ascertainment of first distant brain failure (DBF), death, or censoring. Patients were excluded if active infection or fever, hematologic disease, or autoimmune disease was documented during the pretreatment assessment. Documented corticosteroid, anti-inflammatory, or hematopoietic growth-factor exposure within 14 days before GKRS also constituted an exclusion criterion. No additional exclusionary washout period was imposed for systemic chemotherapy or extracranial radiotherapy. Previous whole-brain radiotherapy (WBRT) or intracranial surgery did not preclude inclusion, and subsequent treatment after GKRS was recorded during follow-up.

The study was approved by the Biomedical Ethics Review Committee of West China Hospital, Sichuan University, on 6 August 2026 (approval no. 1838), including approval for retrospective medical-record research and waiver of research informed consent. All research data collection, medical-record extraction, database preparation, and statistical analyses began after approval. Written informed consent for GKRS treatment was obtained separately. The study was reported in accordance with the Strengthening the Reporting of Observational Studies in Epidemiology (STROBE) statement.

### 2.2. Clinical Variables and Inflammatory Indices

Clinical, pathological, laboratory, and treatment information was retrieved from institutional medical records. Baseline variables included age, Karnofsky Performance Status (KPS), neurological symptoms, molecular subtype, extracranial metastasis, previous WBRT or intracranial surgery, total number of brain metastases, and cumulative treated tumor volume. Total brain-metastasis count included all lesions identified before first GKRS, whereas cumulative treated tumor volume represented the sum of the planned volumes of lesions treated during the index course.

Records were abstracted in two record-abstraction batches by two collectors, comprising 69 and 22 patients. Their first-GKRS dates spanned July 2021–December 2024 and January–June 2025, respectively. These batches belonged to the same institutional cohort and overlapped with treatment period. Batch adjustment assessed sensitivity to collector-associated differences, which could not be separated from calendar-period differences.

Estrogen receptor, progesterone receptor, and human epidermal growth factor receptor 2 (HER2) status were obtained from pathological records. Tumors were classified as HER2-positive, hormone receptor-positive/HER2-negative, or triple-negative. HER2-positive tumors were grouped together irrespective of hormone receptor status.

One blood-count record was available for each patient. All samples were collected before the first GKRS irradiation, including samples collected on the same calendar day, for which the sequence was confirmed by clinical review. Actual sampling dates were retained, and the interval between blood collection and GKRS was calculated. No fixed maximum sampling-to-GKRS interval was imposed.

Blood counts were measured in the clinical laboratory of West China Hospital using automated hematology analyzers under institutional quality-control procedures. Absolute neutrophil, lymphocyte, monocyte, and platelet counts were expressed in ×10^9^/L. The neutrophil-to-lymphocyte ratio (NLR) was calculated as neutrophil count divided by lymphocyte count; platelet-to-lymphocyte ratio (PLR) as platelet count divided by lymphocyte count; lymphocyte-to-monocyte ratio (LMR) as lymphocyte count divided by monocyte count; and systemic immune-inflammation index (SII) as platelet count multiplied by neutrophil count and divided by lymphocyte count. These pretreatment measurements were also used in analyses of outcomes after DBF.

### 2.3. Gamma Knife Radiosurgery

GKRS was performed using a Leksell Gamma Knife Icon system and Leksell GammaPlan version 11.1.1 (Elekta, Stockholm, Sweden). Contrast-enhanced T1-weighted MRI was the principal sequence for target delineation, supplemented by T2-weighted or fluid-attenuated inversion recovery images when available. Targets were delineated by an experienced Gamma Knife neurosurgeon and reviewed by the institutional GKRS team. Treatment plans were assessed for target coverage, isodose distribution, and normal-tissue safety before irradiation.

Prescription dose and fractionation were individualized according to lesion size, number, anatomical location, proximity to critical structures, and normal-tissue tolerance. Planned treatment delivered over multiple sessions was considered part of the same index GKRS course. Subsequent repeat or salvage GKRS was recorded separately.

Lesion-specific target volumes, prescription margin doses, and fractionation schedules were obtained from treatment records. Biologically effective dose was calculated using the linear-quadratic model with an α/β ratio of 10 Gy:

$${\mathrm{BED}}_{10}=\sum_{i=1}^{n}d_{i}\left(1+\frac{d_{i}}{10}\right),$$

where *d_i_* denotes the prescription margin dose delivered in session *i*. The equivalent single-fraction dose was calculated as:

$$D_{\mathrm{eq}}=\frac{\sqrt{100+40\;{\mathrm{BED}}_{10}}-10}{2}.$$

These calculations assumed sufficient repair between planned sessions. Patient-level volume-weighted dose summaries were calculated by weighting each lesion-specific dose by its treated volume and dividing by the cumulative treated volume. The volume-weighted equivalent single-fraction dose was used in the principal adjusted analyses, with the lowest lesion-specific equivalent dose examined in sensitivity analyses. Equivalent doses were treated as comparative dosimetric measures, recognizing the limitations of the linear-quadratic model at high doses per fraction.

### 2.4. Follow-Up and Outcome Definitions

Follow-up information was obtained from available brain MRI examinations, outpatient and inpatient records, and telephone follow-up. Intracranial events were identified from imaging and clinical documentation, and vital status was established from medical records and follow-up information. Brain MRI was routinely recommended at 3 and 6 months after first GKRS and every 6 months thereafter through year 3. Earlier imaging was advised for persistent or concerning neurological symptoms. Actual examination timing varied.

The primary endpoint was first DBF after GKRS, defined as the development of a new brain parenchymal metastasis outside the initially treated targets. The first imaging-documented DBF date was used as the event date. Progression of an initially treated lesion was classified separately as local failure, and isolated leptomeningeal disease did not meet the definition of DBF.

Death before documented DBF was treated as a competing event. Patients who remained alive without documented DBF were censored at the earlier of the last date on which DBF-free status could be confirmed and the administrative study cutoff of 1 July 2026. For patients whose radiographic surveillance extended beyond the administrative cutoff, follow-up was truncated on 1 July 2026.

The multistate framework comprised three states: alive without DBF, alive after DBF, and death. The permitted transitions were from alive without DBF to DBF or death, and from alive after DBF to death. Death was an absorbing state. Post-DBF survival was measured from the first DBF date to death from any cause. Patients remaining alive after DBF were censored at their last confirmed date alive or the administrative cutoff, whichever occurred first.

### 2.5. Clinical Assessment and Treatment After DBF

For patients who developed DBF, KPS and extracranial disease status were recorded at the time of DBF and distinguished from their baseline measurements. Progressive extracranial disease at DBF was included as a covariate in post-DBF survival analyses. The number of extracranial metastatic sites and the systemic treatment line recorded at DBF were used in supplementary adjustment analyses.

Subsequent treatment records were reviewed for systemic treatment initiation or modification, salvage GKRS, WBRT, and intracranial surgery. Actual treatment dates were retained. Systemic treatment exposure represented a documented initiation or change after DBF; absence of this event did not imply that the patient had never received systemic therapy.

Salvage GKRS and systemic treatment initiation or modification were evaluated separately in the principal treatment analyses. WBRT and intracranial surgery were described.

### 2.6. Statistical Analysis

Continuous variables were summarized as medians with interquartile ranges, and categorical variables as counts and percentages using available nonmissing observations. Analyses were conducted at the patient level. NLR, PLR, SII, and LMR were log_2_-transformed and standardized using the mean and sample standard deviation of the full 91-patient cohort. These parameters were retained in analyses restricted to patients with DBF and in resampling analyses. Each inflammatory index was evaluated in a separate model.

Regression analyses used complete observations for the variables required by each model, with a common complete-case set for the four parallel first-DBF models. All patients had complete data for the principal models. Missing values and unknown codes were not treated as negative findings. Follow-up times were analyzed in days, with one month defined as 365.25/12 days for presentation.

#### 2.6.1. First DBF and Competing-Risk Analysis

Cumulative incidence functions were estimated for first DBF and death before DBF. Pointwise 95% confidence intervals (CIs) were calculated from the Aalen variance using a log–log transformation, and cumulative incidence estimates were summarized at 6, 12, and 24 months.

Univariable Fine–Gray models evaluated unadjusted associations with first DBF. Four parallel multivariable models evaluated NLR, PLR, SII, and LMR separately, adjusting for total baseline brain-metastasis count, age, molecular subtype, extracranial metastasis, prior WBRT, and volume-weighted equivalent single-fraction dose. Covariates were not selected according to univariable *p* values. HER2-positive disease was the reference molecular subtype.

Associations were reported as subdistribution hazard ratios (sHRs) with 95% CIs. Effects were expressed per doubling of brain-metastasis count, per 10-year increase in age, per 2 Gy increase in equivalent dose, and per one-standard-deviation increase in each log_2_-transformed inflammatory index. Cause-specific Cox models using the same adjustment set provided complementary estimates of associations with first DBF.

Standardized DBF risk curves were derived from the fitted NLR Fine–Gray model at the 25th, 50th, and 75th percentiles of pretreatment NLR. For each representative value, NLR was assigned that value for every patient while other covariates were retained at their observed values. Individual cumulative incidence predictions were then averaged across the cohort. These curves were presented as point estimates and did not represent observed patient subgroups or clinical thresholds.

#### 2.6.2. Multistate and Post-DBF Survival Analyses

State occupation probabilities were estimated nonparametrically using the Aalen–Johansen estimator and reconstructed patient event histories. Separate cause-specific Cox models examined associations with first DBF, death before DBF, and death after DBF.

Models for death before DBF included all 91 patients from first GKRS, with patients leaving the risk set when DBF occurred. Each inflammatory-marker association was adjusted for age and baseline KPS. Models for death after DBF included the 57 patients who developed DBF, reset time to the DBF date, and adjusted each marker association for age, KPS at DBF, and progressive extracranial disease at DBF. KPS effects were expressed per 10-point increase. Cox models used Efron’s method for tied event times.

Transition-specific estimates were interpreted within their respective risk sets. Differences in statistical significance between transitions were not interpreted as evidence of differences in marker effects. Post-DBF survival was also described using Kaplan–Meier estimates with pointwise log–log 95% CIs.

Salvage GKRS and systemic treatment initiation or modification were evaluated in separate Cox models as time-dependent exposures. Exposure status changed from 0 to 1 at the recorded treatment date and remained 1 thereafter, representing treatment initiation rather than continuous treatment use. Treatment initiated on the DBF date was coded as exposed from the start of post-DBF follow-up.

The treatment models adjusted for pretreatment standardized log_2_ NLR, age, KPS at DBF, progressive extracranial disease at DBF, and a KPS-by-log-time interaction. Conventional model-based variances were used for the principal estimates. Treatment estimates were interpreted as observational associations, with potential residual confounding by treatment selection.

#### 2.6.3. Model Assessment and Sensitivity Analyses

Proportional hazards assumptions for constant-coefficient Cox models were assessed using scaled Schoenfeld residuals and term-specific and global tests. Fine–Gray residuals were examined separately. Exploratory models evaluated time-varying marker associations with first DBF and time-varying coefficients for NLR and KPS after DBF.

Potential nonlinear associations of NLR with first DBF and post-DBF mortality were examined using three-knot restricted cubic splines. The post-DBF spline model included the KPS-by-log-time interaction.

Stability of the first-DBF models was assessed using 1000 patient-level bootstrap samples drawn with replacement. Leave-one-patient-out analyses assessed the influence of individual patients on the estimates.

Sensitivity analyses examined additional adjustment for record-abstraction batch, GKRS year, or baseline KPS; alternative dose and lesion-count measures; and restricted patient subsets. Post-DBF sensitivity analyses examined time since GKRS with delayed entry at DBF and additional molecular-subtype adjustment. Supplementary analyses assessed the association between NLR and first DBF after adjustment for updated Breast-GPA in 90 patients with available scores. Post-DBF mortality analyses additionally adjusted for extracranial metastatic-site count or treatment line in separate models. Detailed specifications for model assessment and sensitivity analyses are provided in the Supplementary Methods.

First DBF was the primary association analysis, post-DBF mortality was secondary, and death before DBF and extended models were exploratory. The cohort size was determined by eligible records without a formal prospective sample-size calculation. Model parameter counts and events per parameter are summarized in Table S10D.

All tests were two-sided, and regression estimates were reported with Wald 95% CIs unless otherwise specified. Benjamini–Hochberg false-discovery-rate adjustment was applied separately to the four adjusted inflammatory-marker tests for first DBF, the eight marker tests across the two mortality transitions, and the two principal time-dependent treatment tests. Additional correction families and analyses reported with nominal *p* values are specified in the Supplementary Methods.

Analyses were performed using R version 4.6.1 (R Foundation for Statistical Computing, Vienna, Austria), with the survival (version 3.8-6), cmprsk (version 2.2-12), and Hmisc (version 5.3-0) packages.

## 3. Results

### 3.1. Patient Characteristics and Observed Outcomes

**Figure 1:**
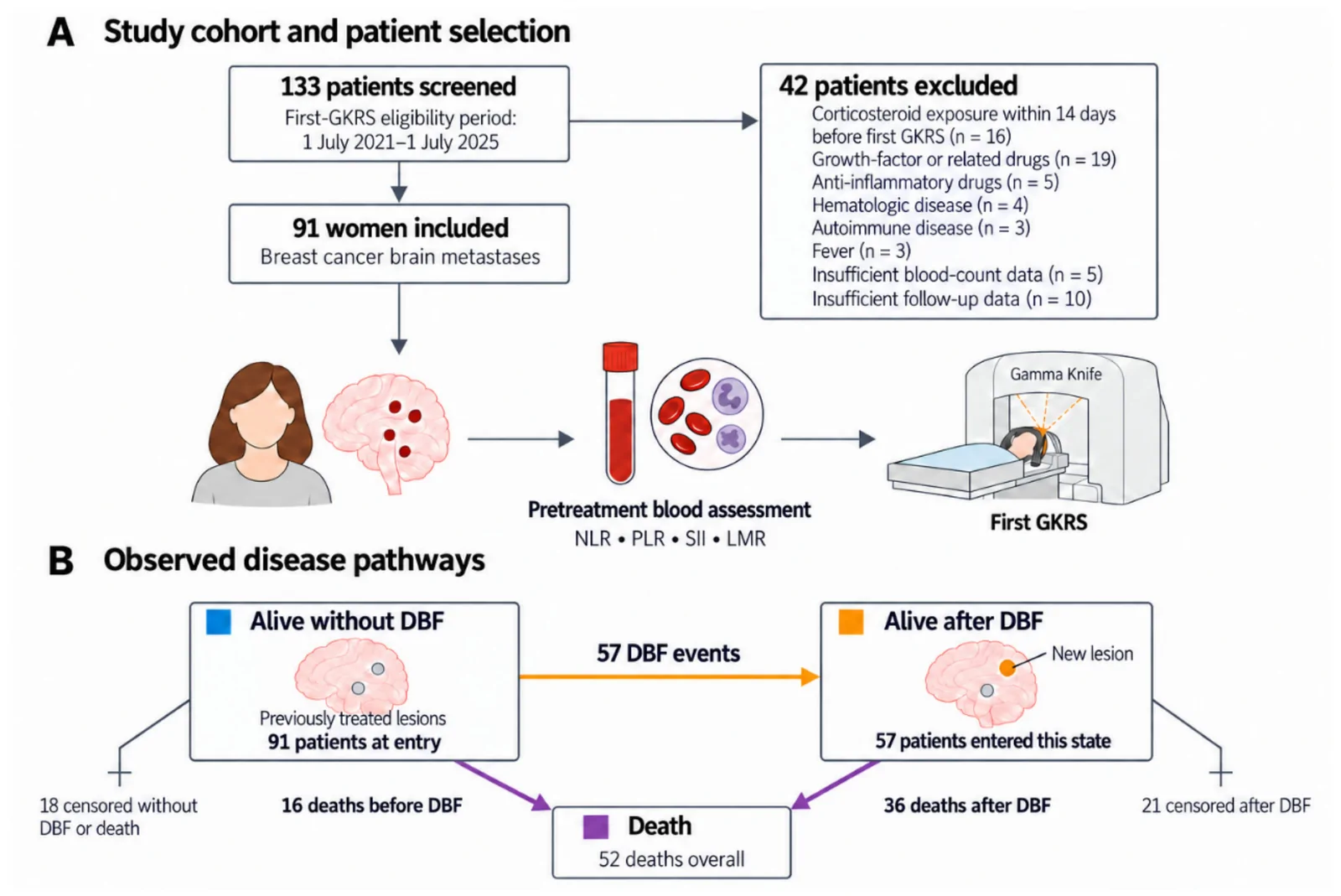
Patient selection and observed disease pathways. (**A**) The institutional screening register identified 133 study candidates for first GKRS between 1 July 2021 and 1 July 2025; 42 were excluded and 91 women with breast cancer brain metastases were included. Exclusion reasons could overlap, so the total number of reasons exceeded the number of excluded patients. Blood sampling preceded irradiation, and the date of first Gamma Knife radiosurgery (GKRS) defined time zero. (**B**) Observed transitions from alive without distant brain failure (DBF; blue) to alive after DBF (orange) or death (purple). There were 57 first-DBF events, 16 deaths before DBF, and 36 deaths after DBF, yielding 52 deaths overall. Eighteen patients were censored while alive without DBF and 21 while alive after DBF. Arrows indicate observed event counts during follow-up, with censoring shown separately. NLR, neutrophil-to-lymphocyte ratio; PLR, platelet-to-lymphocyte ratio; SII, systemic immune-inflammation index; LMR, lymphocyte-to-monocyte ratio.

Of 133 patients screened for the study, 42 were excluded and 91 women were included (Figure 1A). Sixteen excluded patients had documented corticosteroid exposure within the preceding 14 days; exclusion reasons could overlap (Table S10A). Median age was 53 years (interquartile range [IQR], 43.5–59.0), and median baseline Karnofsky performance status (KPS) was 90 (IQR, 85–100). HER2-positive, hormone receptor-positive/HER2-negative, and triple-negative disease accounted for 68.1%, 12.1%, and 19.8% of the cohort, respectively. Extracranial metastases were present in 63 patients (69.2%). Seventeen patients (18.7%) had previously received whole-brain radiotherapy (WBRT), and 16 (17.6%) had undergone craniotomy.

The median baseline brain-metastasis count was 2 (IQR, 1–4.5), with a median of 2 lesions treated at first GKRS (IQR, 1–3). Median cumulative treatment volume was 1.72 cm^3^ (IQR, 0.51–4.22), and the median volume-weighted equivalent single dose was 18.5 Gy (IQR, 16.7–20.0). Prior systemic treatment was recorded for all patients. At GKRS, 28 patients had a recorded HER2 tyrosine kinase inhibitor (TKI)-containing regimen and 18 had a HER2 antibody–drug conjugate (ADC)-containing regimen; these categories overlapped (Table S10C). Median pretreatment NLR, PLR, SII, and LMR were 3.12, 161, 454, and 2.78, respectively. Detailed baseline characteristics are presented in Table 1.

Blood was collected a median of 1 day before GKRS (IQR, 1–1; range, 0–6 days), with all samples obtained before irradiation. Follow-up MRI dates within the first-event observation window were available for 86 patients. The median interval from GKRS to the first recorded follow-up MRI was 77.5 days (IQR, 47.5–117.3). Among 72 patients with at least two examinations, the median interval between examinations, calculated first within each patient and then across patients, was 101.8 days (IQR, 77.0–141.8) (Table S10B).

During follow-up, 57 patients developed DBF and 16 died before DBF. The remaining 18 patients had neither event and were censored without documented DBF. Of these, 15 continued to undergo brain MRI surveillance beyond the administrative cutoff, with no subsequent DBF detected, and were therefore administratively censored on 1 July 2026. The other three patients were censored at their last evaluable DBF-free brain MRI before the cutoff. Among the 57 patients who developed DBF, 36 subsequently died and 21 were censored. Overall, 52 deaths were observed. Figure 1 summarizes the cohort and observed disease pathways.

### 3.2. Cumulative Incidence of DBF and Disease-State Occupation

**Figure 2:**
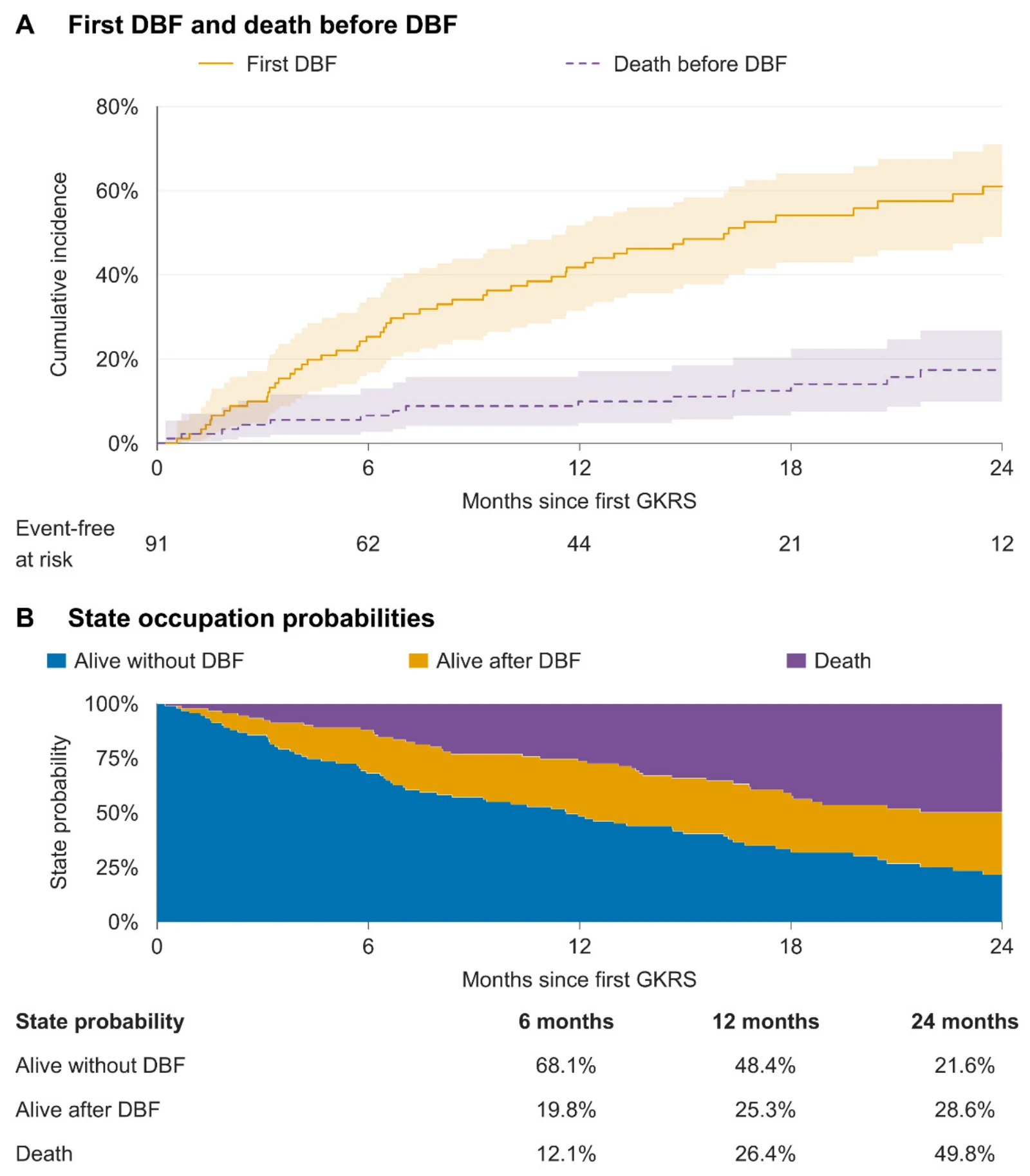
Cumulative incidence and disease-state probabilities after GKRS. (**A**) Cumulative incidence of first distant brain failure (DBF; orange) and death before DBF (purple) in 91 patients. Shading indicates pointwise 95% confidence intervals calculated using Aalen variance and a log–log transformation. The risk table shows patients still under observation without either first event immediately before each time point. (**B**) Aalen–Johansen estimates of the probabilities of being alive without DBF (blue), alive after DBF (orange), or dead (purple). The stacked curves and probability table show point estimates; death includes events before and after DBF. The probability of being alive after DBF is distinct from the cumulative incidence of DBF. Time is measured from first Gamma Knife radiosurgery (GKRS). Both panels display the first 24 months. Numerical estimates at 6, 12, and 24 months are reported in (Table S1), and complete-follow-up curves are shown in (Figure S1).

The cumulative incidence of first DBF was 25.3% (95% CI, 16.8–34.6) at 6 months, 41.8% (95% CI, 31.5–51.7) at 12 months, and 61.0% (95% CI, 49.0–71.0) at 24 months after GKRS. The corresponding cumulative incidences of death before DBF were 6.6% (95% CI, 2.7–13.0), 9.9% (95% CI, 4.8–17.1), and 17.4% (95% CI, 9.8–26.7), respectively (Figure 2A; Table S1A).

At 12 months, the Aalen–Johansen probabilities of being alive without DBF, alive after DBF, and dead were 48.4%, 25.3%, and 26.4%, respectively. By 24 months, these probabilities were 21.6%, 28.6%, and 49.8% (Figure 2B; Table S1B). Complete-follow-up curves are shown in Figure S1.

### 3.3. Clinical Factors and Inflammatory Markers Associated with First DBF

In univariable Fine–Gray analyses, higher baseline brain-metastasis count, extracranial metastasis, and higher pretreatment NLR were associated with first DBF. The remaining clinical variables did not reach nominal statistical significance (Table 2).

**Table 2:** Univariable and multivariable Fine–Gray analyses of factors associated with first distant brain failure.

Variable	Univariable sHR (95% CI)	*p* Value	Adjusted sHR (95% CI)	*p* Value
Age, per 10-year increase	0.94 (0.73–1.22)	0.658	1.16 (0.90–1.51)	0.257
Baseline KPS, per 10-point increase	1.02 (0.87–1.18)	0.832	-	-
Baseline brain-metastasis count, per doubling	1.32 (1.08–1.62)	0.007	1.27 (1.07–1.51)	0.005
Cumulative treatment volume, per doubling	1.02 (0.92–1.13)	0.721	-	-
**Molecular subtype, overall test ^a^**	-	0.227	-	0.358
HER2-positive	1.00 (reference)	-	1.00 (reference)	-
Hormone receptor-positive/HER2-negative	1.92 (0.84–4.41)	0.124	2.02 (0.77–5.27)	0.153
Triple-negative	0.78 (0.36–1.71)	0.540	1.03 (0.42–2.53)	0.954
Extracranial metastases, yes vs. no	2.27 (1.22–4.24)	0.010	2.03 (0.98–4.17)	0.056
Prior WBRT, yes vs. no	0.64 (0.33–1.26)	0.197	0.40 (0.18–0.90)	0.027
Prior craniotomy, yes vs. no	1.39 (0.75–2.59)	0.301	-	-
Baseline neurological symptoms, yes vs. no	0.98 (0.57–1.67)	0.937	-	-
Volume-weighted equivalent single dose, per 2 Gy increase	0.89 (0.78–1.01)	0.073	0.89 (0.74–1.06)	0.178
Pretreatment NLR, per 1 SD increase in log_2_-transformed NLR	1.76 (1.25–2.47)	0.001	1.52 (1.09–2.10)	0.012

**Notes:** All models included 91 patients, with 57 first-DBF events, 16 deaths before DBF, and 18 patients censored without either event. Follow-up began at first GKRS, with death before DBF treated as a competing event. The multivariable model included age, baseline brain-metastasis count, molecular subtype, extracranial metastases, prior WBRT, volume-weighted equivalent single dose, and pretreatment NLR. Covariate inclusion was not based on univariable *p* values. Brain-metastasis count and treatment volume were log_2_-transformed. NLR was log_2_-transformed and standardized using the mean and sample SD of the full cohort. Equivalent single dose was calculated using an α/β ratio of 10 Gy. All *p* values are two-sided and nominal; the multiplicity-adjusted result for NLR is reported in Table 3. Dashes indicate that an estimate or test is not applicable or that the variable was not included in the multivariable model. ^a^ Overall molecular-subtype *p* values were obtained from two-degree-of-freedom Wald tests. CI, confidence interval; DBF, distant brain failure; GKRS, Gamma Knife radiosurgery; HER2, human epidermal growth factor receptor 2; KPS, Karnofsky performance status; NLR, neutrophil-to-lymphocyte ratio; SD, standard deviation; sHR, subdistribution hazard ratio; WBRT, whole-brain radiotherapy.

**Table 3:** Associations of pretreatment inflammatory markers with first distant brain failure.

Marker	Univariable sHR (95% CI)	*p* Value	Adjusted sHR (95% CI)	*p* Value	FDR-Adjusted q Value
NLR	1.76 (1.25–2.47)	0.001	1.52 (1.09–2.10)	0.012	0.047
PLR	1.69 (1.31–2.19)	<0.001	1.38 (1.04–1.83)	0.023	0.047
SII	1.68 (1.24–2.26)	<0.001	1.37 (0.99–1.90)	0.055	0.073
LMR	0.74 (0.54–1.01)	0.059	0.79 (0.60–1.05)	0.106	0.106

**Notes:** Fine–Gray analyses included 91 patients, with 57 first-DBF events and 16 competing deaths; follow-up began at first GKRS. Marker effects are per 1 SD increase in log_2_-transformed values, standardized using the full-cohort mean and sample SD. Each marker was entered into a separate model adjusted for baseline brain-metastasis count, age, molecular subtype, extracranial metastases, prior WBRT, and volume-weighted equivalent single dose. NLR estimates are also shown in Table 2. *p* values are nominal; q values were calculated using the Benjamini–Hochberg procedure across the four adjusted marker tests. CI, confidence interval; DBF, distant brain failure; FDR, false discovery rate; GKRS, Gamma Knife radiosurgery; LMR, lymphocyte-to-monocyte ratio; NLR, neutrophil-to-lymphocyte ratio; PLR, platelet-to-lymphocyte ratio; SD, standard deviation; sHR, subdistribution hazard ratio; SII, systemic immune-inflammation index; WBRT, whole-brain radiotherapy.

In the multivariable model, each 1 SD increase in log_2_-transformed NLR was associated with an adjusted subdistribution hazard ratio (sHR) of 1.52 (95% CI, 1.09–2.10; *p* = 0.012). Baseline brain-metastasis count also remained associated with DBF (sHR per doubling, 1.27; 95% CI, 1.07–1.51; *p* = 0.005). Full clinical-covariate estimates are reported in Table 2.

**Figure 3:**
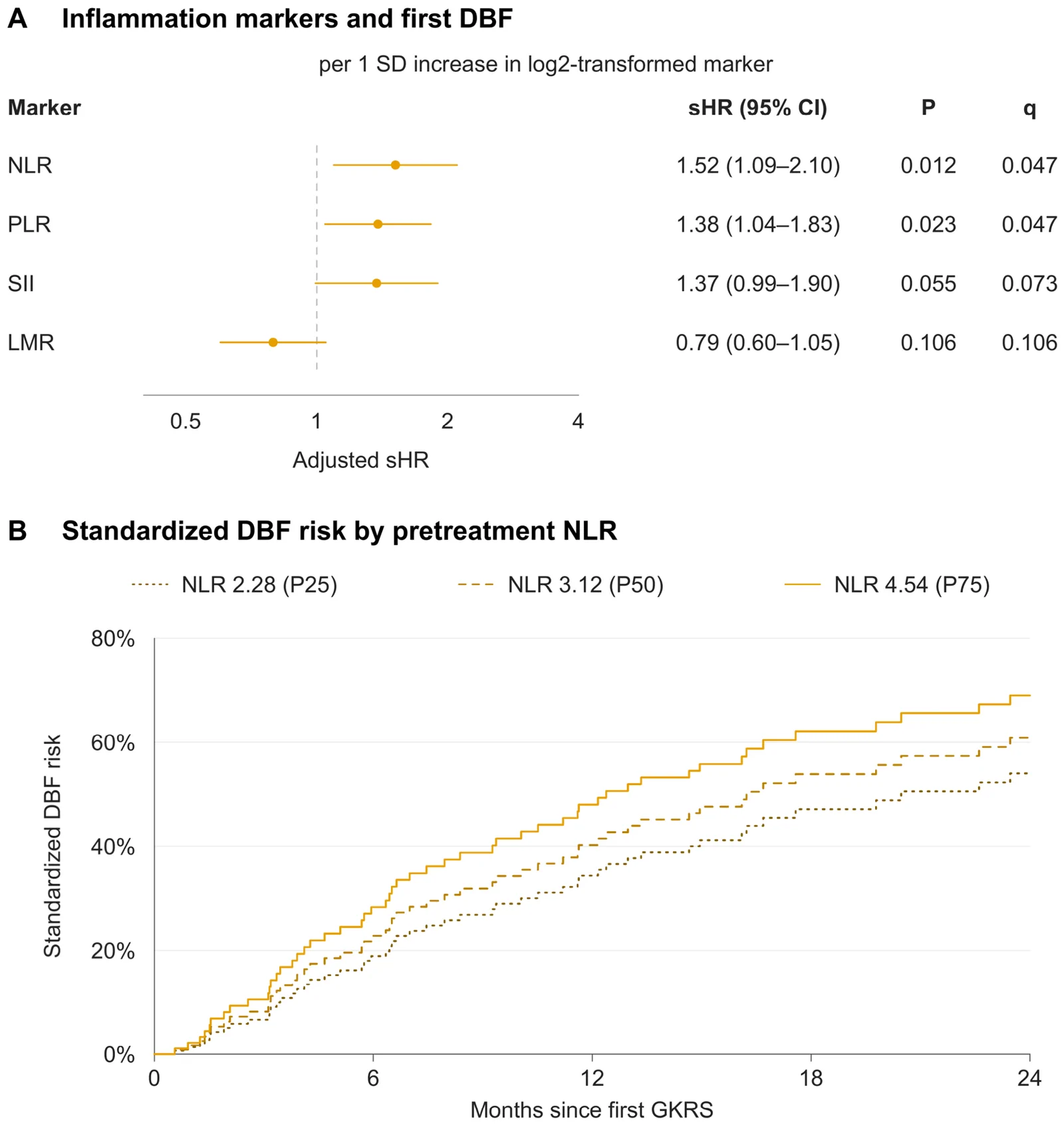
Pretreatment inflammatory markers and first DBF. (**A**) Adjusted Fine–Gray subdistribution hazard ratios (sHRs) with 95% confidence intervals per 1 SD increase in each log_2_-transformed marker. In Panel A, the vertical dashed line indicates sHR = 1. Separate models adjusted for baseline brain-metastasis count, age, molecular subtype, extracranial metastases, prior whole-brain radiotherapy, and volume-weighted equivalent single dose. The q values reflect Benjamini–Hochberg correction across the four adjusted marker tests. (**B**) Standardized cumulative incidence of distant brain failure (DBF) at pretreatment NLR values of 2.28, 3.12, and 4.54, corresponding to the cohort 25th, 50th, and 75th percentiles. In Panel B, dotted, dashed, and solid lines correspond to NLR values of 2.28, 3.12, and 4.54, respectively. For each value, individual predictions from the fitted Fine–Gray model were averaged across all 91 patients, retaining their other observed covariates and the original transformation and standardization parameters. Curves show point estimates at representative NLR values, not observed patient groups or clinical thresholds. Time is measured from first Gamma Knife radiosurgery. NLR, neutrophil-to-lymphocyte ratio; PLR, platelet-to-lymphocyte ratio; SII, systemic immune-inflammation index; LMR, lymphocyte-to-monocyte ratio; SD, standard deviation.

In the parallel inflammatory-marker analyses, NLR and PLR were associated with DBF after BH-FDR correction, with q = 0.047 for both. The adjusted sHR for PLR was 1.38 (95% CI, 1.04–1.83; *p* = 0.023). SII and LMR were not statistically significant after adjustment: the respective sHRs were 1.37 (95% CI, 0.99–1.90; *p* = 0.055; q = 0.073) and 0.79 (95% CI, 0.60–1.05; *p* = 0.106; q = 0.106) (Table 3; Figure 3A). Standardized cumulative incidence curves at NLR values of 2.28, 3.12, and 4.54, corresponding to the cohort quartiles, showed progressively higher estimated DBF incidence with increasing NLR (Figure 3B).

### 3.4. Sensitivity and Supplementary Analyses of First DBF

In cause-specific Cox models, the adjusted hazard ratios were 1.97 for NLR (95% CI, 1.40–2.76; *p* < 0.001), 1.46 for PLR (95% CI, 1.05–2.04; *p* = 0.026), 1.62 for SII (95% CI, 1.16–2.27; *p* = 0.005), and 0.68 for LMR (95% CI, 0.49–0.94; *p* = 0.020) (Table S2A; Figure S2).

Across 1000 patient-level bootstrap resamples, median sHRs were 1.65 for NLR (95% percentile interval, 1.06–2.55) and 1.41 for PLR (1.02–2.10). The percentile intervals for SII and LMR included 1. In the NLR model, the bootstrap median sHR for baseline brain-metastasis count was 1.29 per doubling (95% percentile interval, 1.04–1.87) (Table S3A; Figure S3A).

Adjusted NLR estimates ranged from 1.50 to 1.69 across the eight sensitivity analyses. Additional adjustment for baseline KPS yielded an sHR of 1.60 (95% CI, 1.18–2.18; *p* = 0.003; q = 0.011). Adjustment for record-abstraction batch yielded an sHR of 1.51 (95% CI, 1.09–2.10; *p* = 0.014; q = 0.054), compared with q = 0.047 in the primary analysis. Full results for all four markers are provided in Table S3B1–B8 and Figure S3B; leave-one-patient-out estimate ranges are shown in Figure S3C.

For the NLR restricted cubic spline model, the nonlinear component was not statistically significant (*p* = 0.298), and the overall two-degree-of-freedom test yielded *p* = 0.057 (Table S4; Figure S4A).

Exploratory Fine–Gray time-interaction analyses found no clear evidence of time-varying marker associations (all interaction *p* ≥ 0.189; all q ≥ 0.694; Table S5). Among 90 patients with an originally recorded updated Breast-GPA (56 DBF events and 16 competing deaths), the GPA-adjusted NLR sHR was 1.74 (95% CI, 1.23–2.46; nominal *p* = 0.002; Table S11).

### 3.5. Mortality Before and After DBF

For death before DBF, the adjusted NLR HR was 1.46 (95% CI, 0.78–2.73; *p* = 0.234). Neither age nor baseline KPS was statistically significant in this model, and none of the four inflammatory markers met the FDR threshold for this transition (Table 4; Table S6A; Figure S2). Proportional-hazards testing yielded *p* = 0.003 for the global test and *p* = 0.014 for baseline KPS in the NLR model (Table S6B). These constant-coefficient estimates were exploratory.

**Figure 4:**
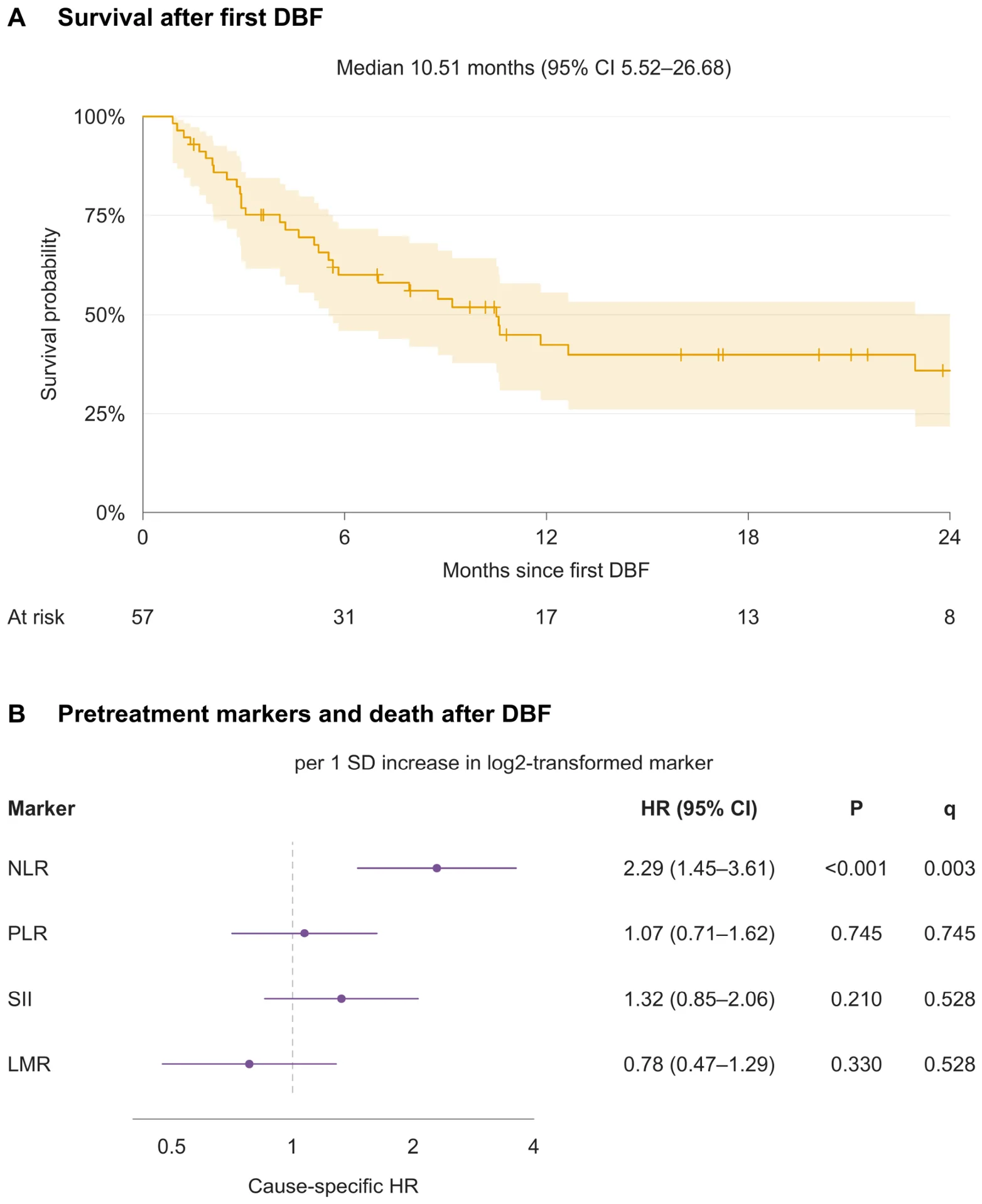
Survival after DBF and associations with pretreatment inflammatory markers. (**A**) Kaplan–Meier survival from first distant brain failure (DBF) in 57 patients, with 36 deaths. Shading indicates pointwise 95% confidence intervals calculated using Greenwood variance and a log–log transformation; plus signs indicate censoring. Numbers at risk are shown below. The median and confidence interval use complete follow-up, although the panel displays 24 months; the full curve is shown in Figure S5. (**B**) Adjusted hazard ratios and 95% confidence intervals from separate constant-coefficient Cox models, with time reset at DBF. The vertical dashed line indicates HR = 1. Each model included age, Karnofsky performance status (KPS) at DBF, progressive extracranial disease at DBF, and one inflammatory marker. Marker effects are per 1 SD increase in log_2_-transformed values, standardized using all 91 patients. All markers were measured before first Gamma Knife radiosurgery. The q values reflect Benjamini–Hochberg correction across eight marker tests covering both mortality transitions. Analyses allowing time-varying coefficients are described in Section 3.5. NLR, neutrophil-to-lymphocyte ratio; PLR, platelet-to-lymphocyte ratio; SII, systemic immune-inflammation index; LMR, lymphocyte-to-monocyte ratio; SD, standard deviation.

Among patients who developed DBF, median survival from DBF was 10.5 months (95% CI, 5.5–26.7). Estimated survival was 60.0% (95% CI, 45.8–71.6) at 6 months, 42.3% (95% CI, 28.4–55.6) at 12 months, and 35.8% (95% CI, 21.8–50.1) at 24 months (Figure 4A; Table S7). The complete-follow-up survival curve is provided in Figure S5.

Higher pretreatment NLR, lower KPS at DBF, and progressive extracranial disease at DBF were associated with post-DBF mortality in univariable analyses. In the constant-coefficient multivariable model, NLR remained associated with mortality (HR, 2.29; 95% CI, 1.45–3.61; *p* < 0.001), as did KPS at DBF (HR per 10-point increase, 0.59; 95% CI, 0.48–0.73; *p* < 0.001). The adjusted HR for progressive extracranial disease was 1.80 (95% CI, 0.74–4.38; *p* = 0.194) (Table 4). Across the eight marker tests covering both mortality transitions, NLR was the only marker to meet the FDR threshold for post-DBF mortality (q = 0.003). PLR, SII, and LMR were not statistically significant in their respective adjusted models (Figure 4B; Table S6A).

The proportional-hazards test identified a departure for KPS in the post-DBF NLR model (*p* = 0.008), but not for NLR (*p* = 0.116; Table S6C). In the model with a time-varying KPS coefficient and a constant NLR coefficient, the adjusted NLR HR was 2.75 (95% CI, 1.68–4.51; nominal *p* < 0.001). When both coefficients were allowed to vary, the *p* values for the KPS and NLR time interactions were 0.048 and 0.342, respectively; the joint NLR main-effect and interaction test yielded *p* < 0.001 (Table S8A; Figure S6).

The direction of the NLR association was unchanged after additional molecular-subtype adjustment and analysis on the GKRS time scale with delayed entry at DBF (Table S8B,C). The post-DBF spline model showed an overall association (*p* < 0.001) without clear nonlinearity (*p* = 0.539; Table S4; Figure S4B). In supplementary analyses with a time-varying KPS coefficient, additional adjustment for extracranial metastatic-site count yielded an NLR HR of 2.65 (95% CI, 1.61–4.38; 56 patients, 35 deaths). Adjustment for recorded treatment line yielded an NLR HR of 3.13 (95% CI, 1.77–5.53; 54 patients, 33 deaths); both nominal NLR *p* values were <0.001. Comparisons with reference models fitted to the same patients are reported in Table S8D.

### 3.6. Treatment After DBF and Subsequent Survival

After DBF, 34 of 57 patients (59.6%) underwent salvage GKRS, and 44 (77.2%) initiated or changed systemic treatment. Among patients receiving each treatment, the median interval from DBF was 26.5 days (IQR, 17.8–50.0) for salvage GKRS and 10.5 days (IQR, 5.0–17.5) for systemic treatment initiation or change (Table S9A). Individual treatment and outcome timelines are shown in Figure S7.

Exploratory time-dependent treatment associations are reported in Table S9B and Figure S8.

## 4. Discussion

In this cohort of women with breast cancer brain metastases, higher pretreatment NLR and PLR were associated with first DBF after multiplicity correction, and NLR was also associated with mortality after DBF. The NLR estimates for first DBF were similar across sensitivity analyses, with adjusted sHRs of 1.50–1.69. Its association with post-DBF mortality persisted after adjustment for clinical status at progression and time variation in the KPS effect. A blood measurement obtained before first GKRS may therefore provide prognostic information across the subsequent disease course. Analyzing DBF and mortality before and after DBF separately makes these associations more clinically interpretable than an overall survival endpoint alone.

Our findings are broadly consistent with earlier evidence linking pretreatment NLR to intracranial progression. Zhang et al. reported an association between elevated pre-SRS NLR and DBF in a mixed-primary cohort that included patients with breast cancer [16]. Koh et al. likewise associated baseline NLR with subsequent brain metastases in stage IV non-small-cell lung cancer [21]. The present study extends these observations to a breast cancer-specific cohort using continuous marker values rather than a selected cutoff. Chowdhary et al., however, found poorer overall survival with elevated post-SRS NLR without a statistically significant difference in distant brain recurrence [22]. Their use of post-treatment blood counts, inclusion of corticosteroid-exposed patients, and survival-derived cutoff distinguish that analysis from ours. These differences may contribute to variation across studies, but do not establish that sampling time alone explains the results. Together, these studies suggest that the timing of measurement, population, and specific intracranial endpoint matter when interpreting an inflammatory marker.

The comparison among inflammatory markers further shows that their associations depend on the endpoint and analytical framework. NLR and PLR met the FDR threshold for first DBF, whereas SII and LMR showed nominal associations only in cause-specific Cox models. The latter assess DBF occurrence among patients still alive and DBF-free, rather than cumulative incidence in the presence of competing death [20]. Earlier SII and LMR studies addressed different populations or outcomes and are therefore not directly comparable [18,19]. Within our cohort, PLR was less consistent across sensitivity analyses. Additional adjustment for KPS or record-abstraction batch had little effect on the NLR estimate, although batch adjustment increased the q value from 0.047 to 0.054. The consistency of the effect estimates therefore supports the observed association, but the borderline FDR-adjusted results warrant caution.

The observed incidence of DBF places these associations within a substantial clinical burden. In the multicenter SRS cohort reported by McTyre et al., competing-risk estimates of DBF were similar to ours during the first year but lower at 24 months [23]. Differences in primary cancer, treatment era, prior cranial treatment, and competing mortality may contribute to this pattern. Their association between baseline brain-metastasis count and DBF also agrees in direction with our finding, although the effect units differed. This consistency supports the relevance of initial intracranial burden and the value of considering new brain disease separately from mortality.

The association between pretreatment NLR and post-DBF mortality adds a different perspective to prognosis after intracranial progression. Farris et al. demonstrated the prognostic relevance of brain metastasis velocity among patients who developed DBF after SRS [24]. In our cohort, median post-DBF survival was 10.5 months, and pretreatment NLR remained associated with mortality when the KPS coefficient was allowed to vary over time (HR, 2.75; 95% CI, 1.68–4.51). Further adjustment for extracranial metastatic-site count or treatment line changed the NLR estimate little in the respective complete-case samples. An earlier blood measurement may therefore retain prognostic information alongside clinical status at progression. However, these adjustments do not distinguish intracranial biology from systemic tumor burden or treatment history. The available site counts and recorded treatment lines are relatively coarse measures, and the finding is best interpreted as a conditional prognostic association among patients who developed DBF.

Molecular subtype and treatment background are important to interpretation. Earlier GKRS studies reported subtype-related differences in failure patterns [25]. Cagney et al. found more new brain metastases in TNBC than in hormone receptor-positive/HER2-negative disease, but their earlier cohort frequently received WBRT [26]. Our cohort was predominantly HER2-positive (68.1%), with smaller comparison groups and different systemic treatment options. The absence of a significant adjusted subtype association therefore does not show that subtype is unimportant. Observational radiotherapy cohorts have linked systemic treatment classes to intracranial outcomes [27], while HER2CLIMB and DESTINY-Breast12 provide more directly relevant evidence for modern HER2-directed regimens [9,10]. Although treatment exposure was recorded at GKRS and DBF and subsequent treatment initiation or change was modeled over time, residual confounding by treatment selection remains. Repeated SRS is also offered to selected patients whose lesions remain amenable to focal treatment [28]. The post-DBF treatment analyses are therefore exploratory and do not establish comparative treatment efficacy.

Experimental evidence offers plausible biological explanations for the NLR findings. Neutrophil extracellular traps can facilitate metastatic behavior through CCDC25, although this work primarily involved liver and lung dissemination [29]. In breast cancer brain-metastasis coculture models, CXCR2 inhibition reduced neutrophil recruitment, NET formation, and tumor migration and invasion [30]. Studies of carcinoma–astrocyte communication and inflammatory signaling further connect the brain microenvironment with metastatic growth [31,32]. Platelet depletion in a breast cancer model reduced myeloid-cell infiltration and tumor growth while increasing cytotoxic T-cell infiltration, providing related biological context for PLR [33]. These experiments support the relevance of inflammatory processes, but circulating cell ratios measure neither activation nor tissue-specific immune function. NLR is best viewed here as a clinical correlate of the host–tumor state rather than a direct readout of one mechanism.

These findings identify a readily available candidate marker for further evaluation alongside intracranial burden and clinical status. Continuous modeling and standardized incidence curves describe the association without introducing a data-derived clinical cutoff. The NLR association with first DBF persisted after adjustment for updated Breast-GPA in 90 patients with available scores, but its incremental predictive value and clinical utility remain unproven. The most informative findings concern first DBF and mortality after DBF; differences in statistical significance between transitions do not themselves establish different effects. Independent cohorts and longitudinal blood measurements could clarify whether pretreatment levels or subsequent changes add useful information before NLR is used to alter surveillance or treatment.

Several limitations should be acknowledged. This retrospective, single-center study included a modest number of patients and events, particularly the 16 deaths before DBF, limiting precision and increasing the potential for overfitting. Post-DBF analyses describe prognosis conditional on developing DBF and surviving to that event; multistate modeling and covariate adjustment do not eliminate selection or collider bias. The predominance of HER2-positive disease and heterogeneous systemic treatments limit generalizability and confounding control. Exclusion of corticosteroid-exposed patients may underrepresent symptomatic patients requiring steroids. A single pretreatment blood sample cannot capture evolving inflammatory states, and variable MRI intervals may affect the recorded timing of DBF. For death before DBF, the small event count and evidence of proportional-hazards violations for KPS and the model overall particularly limit interpretation of the constant-coefficient model; nonsignificant marker results should not be read as evidence of no association.

## 5. Conclusions

In patients with breast cancer brain metastases treated with GKRS, higher pretreatment NLR and PLR were associated with a greater cumulative incidence of first DBF. Pretreatment NLR was also associated with mortality after DBF, after adjustment for the clinical factors included in the model. These findings suggest that NLR may capture prognostic information relevant to both intracranial progression and subsequent survival, supporting its further evaluation alongside intracranial disease burden and clinical status. External validation and direct assessment of predictive performance are required before using NLR to guide surveillance or treatment.

## Figures and Tables

**Table 1:** Baseline characteristics of the study cohort.

Characteristic	All Patients (*n* = 91)
**Demographic and clinical characteristics**	
Age, years	53.0 (43.5–59.0)
Female sex	91 (100.0)
Baseline KPS	90 (85–100)
**Molecular subtype**	
HER2-positive	62 (68.1)
Hormone receptor-positive/HER2-negative	11 (12.1)
Triple-negative	18 (19.8)
Neurological symptoms	33 (36.3)
Extracranial metastases	63 (69.2)
Updated Breast-GPA ^a^	3.0 (2.0–3.5)
**Intracranial disease burden**	
Total number of brain metastases	2.0 (1.0–4.5)
Number of lesions treated at first GKRS	2.0 (1.0–3.0)
Cumulative treatment volume, cm^3^	1.72 (0.51–4.22)
**Previous treatment**	
WBRT	17 (18.7)
Craniotomy	16 (17.6)
Any systemic treatment	91 (100.0)
Chemotherapy ^b^	79 (89.8)
Any targeted therapy ^b^	68 (77.3)
HER2-targeted therapy ^b^	54 (61.4)
HER2 tyrosine kinase inhibitor ^b^	37 (42.0)
Endocrine therapy ^b^	26 (29.5)
Immunotherapy ^b^	5 (5.7)
Bone-modifying treatment ^b^	9 (10.2)
**First GKRS treatment parameters**	
Volume-weighted margin dose, Gy	13.0 (12.0–18.0)
Minimum margin dose, Gy	13.0 (12.0–18.0)
Volume-weighted equivalent single dose, Gy	18.5 (16.7–20.0)
Minimum equivalent single dose, Gy	18.0 (15.5–20.0)
**Pretreatment blood assessment**	
Interval from blood collection to first GKRS, days	1 (1–1)
NLR	3.12 (2.28–4.54)
PLR	161 (110–228)
SII	454 (296–920)
LMR	2.78 (2.09–3.94)

**Notes:** Values are median (interquartile range) or *n* (%). Characteristics were assessed before or at first GKRS, and all blood samples were collected before irradiation. Treatment categories may overlap. Equivalent single doses were calculated using an α/β ratio of 10 Gy. Inflammatory markers are reported on their original scales. ^a^ Breast-GPA was available for 90 patients. ^b^ Detailed treatment-class information was available for 88 patients; percentages for these categories use 88 as the denominator. Receipt of any prior systemic treatment was documented for all 91 patients. GKRS, Gamma Knife radiosurgery; GPA, graded prognostic assessment; HER2, human epidermal growth factor receptor 2; KPS, Karnofsky performance status; LMR, lymphocyte-to-monocyte ratio; NLR, neutrophil-to-lymphocyte ratio; PLR, platelet-to-lymphocyte ratio; SII, systemic immune-inflammation index; WBRT, whole-brain radiotherapy.

**Table 4:** Univariable and multivariable Cox analyses of mortality before and after distant brain failure.

(A) Death Before DBF (*n* = 91; 16 Deaths)				
Variable	Univariable HR (95% CI)	*p* Value	Adjusted HR (95% CI)	*p* Value
Age, per 10-year increase	1.35 (0.83–2.19)	0.228	1.29 (0.75–2.21)	0.353
Baseline KPS, per 10-point increase	0.83 (0.66–1.05)	0.124	0.91 (0.69–1.19)	0.493
Pretreatment NLR, per 1 SD increase in log_2_-transformed NLR	1.47 (0.81–2.66)	0.202	1.46 (0.78–2.73)	0.234
**(B) Death After DBF (*n* = 57; 36 Deaths)**				
**Variable**	**Univariable HR (95% CI)**	***p*** **Value**	**Adjusted HR (95% CI)**	***p*** **Value**
Age, per 10-year increase	0.86 (0.59–1.23)	0.400	0.85 (0.56–1.28)	0.428
KPS at DBF, per 10-point increase	0.56 (0.47–0.67)	<0.001	0.59 (0.48–0.73)	<0.001
Progressive extracranial disease at DBF, vs. absent/stable	4.46 (2.12–9.40)	<0.001	1.80 (0.74–4.38)	0.194
Pretreatment NLR, per 1 SD increase in log_2_-transformed NLR	2.53 (1.70–3.77)	<0.001	2.29 (1.45–3.61)	<0.001

**Notes:** Panel A presents cause-specific Cox models with follow-up beginning at first GKRS; patients developing DBF exited the risk set at DBF. The multivariable model included age, baseline KPS, and pretreatment NLR. Panel B presents Cox models with time reset at first DBF; the multivariable model included age, KPS at DBF, progressive extracranial disease at DBF, and pretreatment NLR. NLR was measured before first GKRS in both panels and was log_2_-transformed and standardized using all 91 patients. Models used Efron’s method for ties and model-based variance estimates. All *p* values are two-sided and nominal. These models used constant coefficients. Proportional-hazards testing identified departures for baseline KPS in Panel A (*p* = 0.014; global *p* = 0.003) and KPS at DBF in Panel B (*p* = 0.008). Proportional-hazards diagnostics are reported in (Table S6B,C). Parallel inflammatory-marker mortality analyses and their multiplicity-adjusted results are reported in (Table S6A). In the model with a time-varying KPS coefficient, the post-DBF NLR HR was 2.75 (95% CI, 1.68–4.51; nominal *p* < 0.001; Table S8A). CI, confidence interval; DBF, distant brain failure; GKRS, Gamma Knife radiosurgery; HR, hazard ratio; KPS, Karnofsky performance status; NLR, neutrophil-to-lymphocyte ratio; SD, standard deviation.

## Data Availability

The datasets analyzed during the current study are not publicly available because they contain patient-level clinical information and are subject to institutional ethical restrictions. De-identified data may be available from the corresponding author(s) upon reasonable request and with appropriate institutional and ethics committee approval.

## Supplementary Materials

The following supporting information can be downloaded at: https://www.mdpi.com/article/10.3390/jcm15218701/s1, Supplementary Methods; Table S1: Cumulative incidence of first events and disease-state occupation probabilities; Table S2: Cause-specific Cox analyses of first distant brain failure; Table S3: Bootstrap stability and sensitivity analyses of first distant brain failure; Table S4: Restricted cubic spline analyses of pretreatment NLR; Table S5: Time-varying inflammatory-marker associations with first distant brain failure; Table S6: Inflammatory-marker associations with mortality before and after distant brain failure; Table S7: Survival estimates after distant brain failure; Table S8: Additional analyses of mortality after distant brain failure; Table S9: Treatment timing and time-dependent associations with mortality after distant brain failure; Table S10: Screening, surveillance, treatment characteristics, and model complexity; Table S11: Updated Breast-GPA supplementary association analysis; Figure S1: Cumulative incidence and disease-state probabilities over complete follow-up; Figure S2: Inflammatory-marker associations across three disease transitions; Figure S3: Bootstrap stability, sensitivity analyses, and influence diagnostics; Figure S4: Shape of the association between pretreatment NLR and disease outcomes; Figure S5: Survival after DBF over complete follow-up; Figure S6: Time-varying associations of NLR and KPS with mortality after DBF; Figure S7: Individual treatment and outcome timelines after DBF; Figure S8: Time-dependent treatment initiation and survival after DBF.
