# Supplementary material for: Distant Brain Failure and Subsequent Survival After Gamma Knife Radiosurgery for Breast Cancer Brain Metastases: Prognostic Value of Pretreatment Inflammatory Markers
Source: J Clin Med. 2026 Nov 7;15(21):8701. doi: 10.3390/jcm15218701 (PMC13763871; doi:10.3390/jcm15218701)
Supplement: Supplementary file 1 [file jcm-15-08701-s001.zip › jcm-4614493-supplementary.pdf]

Supplementary material

Distant Brain Failure and Subsequent Survival After Gamma Knife Radiosurgery for Breast Cancer Brain Metastases: Prognostic Value of Pretreatment Inflammatory Markers

Sijun Diao [1], Fujun Liu [2], Zhongyu Li [1], Shihong Zhu [1], Xiyue Lu [3], Huiyang Luo [3] and Jing Chen [1,*]

1. Department of Neurosurgery, West China Hospital, West China Medical School, Sichuan University, Chengdu 610041, China; diaosijun4016@163.com (S.D.); lzainleey@163.com (Z.L.); austin_zhushihong@163.com (S.Z.)

2. State Key Laboratory of Biotherapy and Cancer Center, West China Hospital, Sichuan University , Chengdu 610041, China; fjliu2017@163.com

3. Department of Anesthesiology, West China Hospital, West China Medical School, Sichuan University, Chengdu 610041, China; 15680613113@163.com (X.L.); luohuiyang0216@outlook.com (H.L.)

* Correspondence: jinc@scu.edu.cn

Supplementary Methods

These methods describe the competing-risk, multistate, model-assessment, and treatment analyses in the 91-patient cohort, including the 57 patients who developed distant brain failure (DBF). Supplementary Tables S1–S11 and Figures S1–S8 report the corresponding results.

Analysis populations and variable coding

The first-event analyses included 91 patients, with 57 first-DBF events, 16 deaths before DBF, and 18 observations censored without either event. Post-DBF analyses included all 57 patients who developed DBF, with 36 deaths and 21 censored observations. The date of first GKRS defined time zero for first-event and multistate analyses. Primary post-DBF mortality analyses reset time to the first DBF date. Calendar times were recorded in days; one month was defined as 365.25/12 days. Analyses used model-specific complete cases rather than requiring completeness of every descriptive variable; all principal marker models included the full relevant analysis population. Unknown categorical values were treated as missing, not as absence of the characteristic.

NLR, PLR, SII, and LMR were calculated from the pretreatment blood counts. Each marker was $\log_2$-transformed and standardized using its mean and sample standard deviation in all 91 patients. These parameters were retained in every subgroup, bootstrap, and sensitivity analysis. Age was entered per 10 years, KPS per 10 points, brain-metastasis and treated-lesion counts on the $\log_2$ scale, and equivalent single-fraction dose per 2 Gy. HER2-positive disease was the reference for the three-category molecular subtype; absence was the reference for binary baseline characteristics. Each inflammatory marker was evaluated in a separate model.

First events and state occupation

First DBF and death before DBF were treated as mutually exclusive first events. Cumulative incidence functions were estimated with cmprsk::cuminc. Pointwise 95% confidence intervals used the Aalen variance with a $\log(-\log(F))$ transformation, where F is the estimated cumulative incidence. Estimates at 6, 12, and 24 months were obtained from the right-continuous step functions without interpolation or extrapolation. Event-free risk counts comprised patients still observed without either first event immediately before the stated time.

The multistate model allowed transitions from alive without DBF to alive after DBF or death, and from alive after DBF to death. Aalen–Johansen state occupation probabilities were estimated with survival::survfit using patient identifiers, start–stop intervals, and the state at interval entry. State

probabilities and pointwise log–log confidence intervals were calculated nonparametrically, without a covariate Cox model. The probability of being alive after DBF is not the cumulative incidence of DBF. Kaplan–Meier estimates of post-DBF survival used Greenwood variance with log–log confidence intervals; the median and its interval used complete follow-up. The 24-month display windows did not truncate the analyses.

Regression models and standardized risk curves

Fine–Gray models for first DBF used cmprsk::crr with DBF as the event of interest and death before DBF as the competing event. Each adjusted marker model included $\log_2$ baseline brain-metastasis count, age, molecular subtype, extracranial metastasis, prior WBRT, and volume-weighted equivalent single-fraction dose. The same covariates were used in the complementary first-DBF cause-specific Cox models. Models for death before DBF adjusted each marker association for age and baseline KPS; patients developing DBF left that risk set at DBF. Models for death after DBF adjusted for age, KPS at DBF, and progressive extracranial disease at DBF. Cox models used Efron ties. No covariate selection was based on univariable P values.

Standardized first-DBF risk curves were obtained directly from the fitted NLR Fine–Gray model. Pretreatment NLR was set to its cohort 25th, 50th, or 75th percentile (2.28, 3.12, and 4.54, respectively) for every patient, with all other covariates retained at their observed values. The original $\log_2$ transformation and standardization parameters were applied, individual predicted cumulative incidence functions were calculated, and predictions were averaged over all 91 patients. Only point estimates were plotted. The representative values did not define observed patient groups or clinical thresholds.

Model diagnostics and time varying coefficients

Scaled Schoenfeld residuals and term-specific and global cox.zph tests with the Kaplan–Meier time transformation assessed proportional hazards in constant-coefficient Cox models. Fine–Gray residuals were examined separately; Cox diagnostics were not treated as tests of Fine–Gray proportionality. Model checks also included convergence, matrix rank, finite standard errors, and unusually wide intervals.

For first DBF, each adjusted Fine–Gray model was extended by a marker-by-log-time interaction, $\gamma z \log(t/365.25)$, where z was the standardized $\log_2$ marker and t was the internal event time in days from GKRS. The marker main effect and all clinical covariates were retained. The crr cov2 and tf arguments implemented the interaction at event times. The main-effect coefficient applied at 12 months. A one-degree-of-freedom Wald test assessed $\gamma = 0$, whereas a two-degree-of-freedom Wald test assessed the

joint null $\beta = \gamma = 0$. Time-specific sHRs were $\exp[\beta + \gamma \log(t/365.25)]$; their pointwise intervals used the full covariance matrix. These models and time-specific estimates are reported in Table S5.

After DBF, the constant-coefficient NLR model was extended in turn to allow time variation in KPS, NLR, or both. Interactions used $\gamma x \log(t/182.625)$, with t in days from DBF, so main effects applied at six months. The coxph tt mechanism evaluated the transformation at the event time for every patient in the risk set; joint and interaction-only Wald tests distinguished overall NLR association from evidence of time variation. Pointwise 95% confidence intervals were calculated on the log-hazard scale using the variances and covariance of the main-effect and time-interaction coefficients, then exponentiated. Values were evaluated only at positive times within the observed range. Models with tt terms were not subjected to cox.zph.

Nonlinearity and additional adjustment

NLR restricted cubic splines used three fixed knots at the 10th, 50th, and 90th percentiles of standardized $\log_2$ NLR in all 91 patients. The corresponding NLR values were approximately 1.88, 3.12, and 6.54; the cohort median of 3.12 was the plotting reference. The basis comprised one linear and one nonlinear term. The first-DBF spline model retained the full Fine–Gray adjustment set. The post-DBF spline model retained age, KPS at DBF, progressive extracranial disease, and the KPS-by-log-time interaction, without an NLR-spline-by-time interaction. Overall and nonlinear components were assessed using two- and one-degree-of-freedom Wald tests, respectively, rather than an ordinary likelihood-ratio test of the Fine–Gray pseudolikelihood. Pointwise intervals for curves used basis differences from the reference and the complete covariance matrix.

Post-DBF sensitivity analyses added the two molecular-subtype coefficients to the constant-coefficient NLR model or to the NLR model allowing time variation in KPS. NLR remained linear in these analyses. A separate clock-forward sensitivity analysis retained the original post-DBF adjustment variables but measured time from GKRS, with delayed entry at DBF and exit at DBF time plus subsequent survival time. Time before DBF did not contribute to the post-DBF death risk set. Clock-reset and clock-forward models were reported separately; their coefficients were not combined to construct state probabilities **(Table S8C)**.

Bootstrap and sensitivity analyses

A total of 1,000 patient-level bootstrap samples were drawn with replacement from the 91 patients, each containing 91 records, using seed 20260915. Sampling was not stratified by outcome, duplicate draws

were retained, and the same sample was used for all four marker models. Covariates, reference levels, and full-cohort standardization parameters were held fixed. All 1,000 fits per marker were successful. Summaries comprised the median sHR, 2.5th and 97.5th percentiles, and the proportion of coefficient signs agreeing with the original estimate. Brain-metastasis-count coefficients were also summarized. Each of 91 patients was separately omitted in an influence analysis, with all four models refitted for each omission; plotted ranges were not 95% confidence intervals **(Table S3; Figure S3)**.

Eight sensitivity analyses each changed one component of the first-DBF adjustment framework: (1) additional adjustment for the record-abstraction batch (69 and 22 records), with the 69-record batch as reference; (2) additional adjustment for GKRS calendar year, centered on the full-cohort mean; (3) additional adjustment for baseline KPS per 10 points; (4) replacement of volume-weighted equivalent dose by the lowest equivalent dose, per 2 Gy; (5) replacement of total baseline brain-metastasis count by first-course treated-lesion count, both $\log_2$-transformed; (6) exclusion of prior-WBRT recipients and removal of the resulting constant WBRT covariate; (7) exclusion of patients with >10 baseline brain metastases or a cumulative treated volume >25 cm^3; and (8) exclusion of records requiring adjudication of whether an intracranial event was outside the original treatment target. The first five analyses retained 91 patients and 57 DBF events; the final three included 74/48, 86/52, and 88/55 patients/DBF events, respectively.

The record-abstraction batches comprised 69 patients treated in 2021–2024 and 22 in 2025, collected by two individuals at the same institution. Batch adjustment assessed collector-associated differences but could not separate them from treatment-period differences.

The endpoint-review sensitivity analysis removed three patients whose source records requested determination of whether a suspected intracranial event represented out-of-target DBF. Two had a DBF event in the primary analysis and one was censored. This exclusion tested sensitivity to those endpoint-classification flags; it did not classify all three as confirmed local failures or alter their primary-analysis endpoints. Each sensitivity analysis used common complete cases across the four marker models and retained the original standardization parameters.

Time dependent treatment exposures

Treatment intervals were reconstructed from the DBF date, actual treatment-start dates, and the death or censoring endpoint. Separate exposures described salvage GKRS and systemic treatment initiation or modification. Each changed from 0 before initiation to 1 thereafter and remained 1, denoting that

treatment had been initiated, not continuous use. Start on the DBF date was coded as 1 from the first interval. Under the (start, stop] convention, a death on the same day as treatment initiation would be assigned to the pre-switch state if intraday ordering were unknown; no such same-day death was present. A value of 0 for systemic treatment change did not imply that no systemic therapy had ever been received. Each principal treatment Cox model included the time-dependent exposure, pretreatment standardized $\log_2$ NLR, age per 10 years, KPS at DBF per 10 points, progressive extracranial disease at DBF, and KPS × log(t/182.625), with time in days after DBF. Conventional model-based variance was used for the principal estimates. Results are reported in Table S9B. The two principal treatments were not mutually adjusted, and baseline-at-DBF covariates did not capture all changes influencing treatment selection. Associations were not interpreted as causal effects.

Supplementary adjustment analyses

The regimen recorded at GKRS, the regimen and systemic treatment line recorded at DBF, and extracranial metastatic-site count at DBF were abstracted separately. These time-point records were distinct from prior-treatment history and from post-DBF initiation or change. Unknown information remained missing, and specific drugs were not inferred from historical treatment-class flags. The DBF treatment-line field, including clinically confirmed values, was analyzed per one-line increase; uniform criteria for defining line transitions were not available.

The post-DBF NLR model with a KPS-by-log-time coefficient was separately extended by metastatic-site count or treatment line, each as a continuous covariate. For each extension, a reference model was fitted in exactly the same complete-case patients. All models retained age, KPS at DBF, progressive extracranial disease, the original full-cohort NLR standardization, Efron ties, and log(t/182.625) for the KPS time effect. Site-count analyses included 56 patients with 35 deaths; treatment-line analyses included 54 patients with 33 deaths. Missing values were not imputed. Full coefficients are reported in Table S8D; P values are nominal.

The 2020 updated Breast-GPA used the corrected scoring scheme of Sperduto et al. KPS ≤60, 70–80, or 90–100 contributed 0, 0.5, or 1 point; age <60 years, a single brain metastasis, and absence of extracranial metastases each contributed 0.5 point; HER2-positive, hormone receptor-positive/HER2-negative, and triple-negative disease contributed 1.5, 0.5, and 0 points. Components were the baseline age, KPS, brain-metastasis count, extracranial-metastasis status, and molecular subtype recorded for first GKRS, not measurements at subsequent DBF. Analyses included 90 patients with available

baseline Breast-GPA scores; the missing score was not imputed. NLR-only, GPA-only, and NLR-plus-GPA Fine–Gray models used these same 90 patients, with 56 DBF events, 16 competing deaths, and 18 censored observations (Table S11). The original 91-patient NLR $\log_2$ mean and sample SD were retained. GPA and its individual components were not jointly entered.

MRI interval summaries used recorded examination dates after first GKRS and no later than the first-event endpoint, including the confirmed MRI dates of first DBF. Duplicate dates within a patient were counted once. The first-examination interval was summarized per patient. Consecutive intervals were summarized both across examinations and as each patient's median interval; patients with only one examination contributed only to the first-examination summary. The interval from the preceding recorded MRI to the first-DBF MRI was summarized separately; the preceding scan was not assumed to have been confirmed negative.

Multiplicity and interpretation

Benjamini–Hochberg correction was applied to distinct families: four adjusted marker tests for first DBF; four univariable marker tests for first DBF; four marker-by-time interaction tests for first DBF; four marker tests within each of the eight first-DBF sensitivity analyses; eight marker tests spanning death before DBF and clock-reset death after DBF; and the two principal time-dependent treatment coefficients. These families were not pooled. Clock-forward, spline, subtype-adjustment, post-DBF time-effect, GPA, and additional disease-burden adjustment tests were reported with nominal P values. The correction for one family was not transferred to another model. A difference between significant and nonsignificant transition-specific results was not considered a formal between-transition effect comparison. Analyses used R 4.6.1, survival 3.8-6, cmprsk 2.2-12, and Hmisc 5.3-0.

Table S1. Cumulative incidence of first events and disease-state occupation probabilities

A. Cumulative incidence of first events

Outcome	Months after GKRS	CIF, % (95% CI)	Event-free at risk, n
First DBF	6	25.27 (16.84–34.58)	62
First DBF	12	41.76 (31.49–51.69)	44
First DBF	24	60.96 (48.98–70.95)	12
Death before DBF	6	6.59 (2.68–12.97)	62
Death before DBF	12	9.89 (4.83–17.10)	44

Death before DBF	24	17.39 (9.84–26.73)	12

B. Disease-state occupation probabilities

Months after GKRS	Alive without DBF, % (95% CI)	Alive after DBF, % (95% CI)	Death, % (95% CI)	At risk without DBF, n	At risk after DBF, n
6	68.13 (57.50–76.64)	19.78 (12.35–28.50)	12.09 (6.42–19.68)	62	18
12	48.35 (37.78–58.13)	25.27 (16.89–34.51)	26.37 (17.83–35.69)	44	23
24	21.65 (12.93–31.83)	28.55 (18.73–39.15)	49.80 (38.36–60.21)	12	14

Notes: CIFs and state probabilities were estimated from first GKRS in all 91 patients. One month equals 365.25/12 days. CIF intervals use Aalen variance with a log–log transformation; Aalen–Johansen state probabilities have pointwise log–log intervals. Risk counts refer to observed patients immediately before each time point; panel A shows the event-free risk set, whereas panel B shows the risk sets in the two living states. Death includes both mortality transitions. DBF, distant brain failure; GKRS, Gamma Knife radiosurgery; CIF, cumulative incidence function; CI, confidence interval.

Table S2. Cause-specific Cox analyses of first distant brain failure

A. Cause-specific Cox associations with first DBF

Marker	Patients, n	DBF events, n	Adjusted HR (95% CI)	P value
NLR	91	57	1.97 (1.40–2.76)	<0.001
PLR	91	57	1.46 (1.05–2.04)	0.026
SII	91	57	1.62 (1.16–2.27)	0.005
LMR	91	57	0.68 (0.49–0.94)	0.020

Notes: Each marker was entered into a separate Cox model, with effects per 1-SD increase in log_2-transformed values standardized using all 91 patients. Models adjusted for baseline brain-metastasis count, age, molecular subtype, extracranial metastases, prior WBRT, and volume-weighted equivalent single dose. Models used Efron ties. P values are nominal.

B. Proportional-hazards diagnostics for first-DBF cause-specific Cox models

n = 91; events = 57.

Marker model	Term or test	χ^2	df	Diagnostic P value
NLR	Baseline brain-metastasis count, per doubling	0.03	1	0.867
NLR	Age, per 10 years	2.09	1	0.148

NLR	Molecular subtype (overall)	2.97	2	0.226
NLR	Extracranial metastasis	0.25	1	0.619
NLR	Prior WBRT	1.14	1	0.285
NLR	Volume-weighted equivalent dose, per 2 Gy	0.01	1	0.919
NLR	Pretreatment NLR, per 1-SD $\log_2$ increase	1.93	1	0.165
NLR	Global test	7.39	8	0.495
PLR	Global test	5.33	8	0.722
SII	Global test	4.54	8	0.805
LMR	Global test	6.10	8	0.636

Notes: Scaled Schoenfeld-residual tests used the Kaplan–Meier time transformation. Term-specific and global tests are shown for NLR models; global tests are also shown for parallel PLR, SII, and LMR models. These P values assess proportional hazards rather than covariate associations. Cox diagnostics do not assess the Fine–Gray proportional subdistribution-hazards assumption. DBF, distant brain failure; HR, hazard ratio; CI, confidence interval; SD, standard deviation; KPS, Karnofsky performance status; WBRT, whole-brain radiotherapy; NLR, neutrophil-to-lymphocyte ratio; PLR, platelet-to-lymphocyte ratio; SII, systemic immune-inflammation index; LMR, lymphocyte-to-monocyte ratio; df, degrees of freedom.

Table S3. Bootstrap stability and sensitivity analyses of first distant brain failure

A. Patient-level bootstrap stability

Marker model	Coefficient	Original sHR	Bootstrap median sHR (95% percentile interval)	Direction agreement, %
NLR	Inflammatory marker	1.52	1.65 (1.06–2.55)	98.6
PLR	Inflammatory marker	1.38	1.41 (1.02–2.10)	98.4
SII	Inflammatory marker	1.37	1.43 (0.98–2.14)	97.0
LMR	Inflammatory marker	0.79	0.79 (0.45–1.13)	89.4
NLR	Brain-metastasis count	1.27	1.29 (1.04–1.87)	98.8

PLR	Brain-metastasis count	1.25	1.28 (1.03–1.73)	99.1
SII	Brain-metastasis count	1.27	1.29 (1.05–1.80)	99.4
LMR	Brain-metastasis count	1.23	1.25 (0.99–1.80)	96.9

Notes: All 1,000 patient-level bootstrap fits were successful for each marker model. Resampling was performed at the patient level, with replacement, without stratification. The same resamples were used across marker models; transformations and clinical adjustment sets were retained. Percentile intervals are the 2.5th and 97.5th percentiles of bootstrap estimates. Marker effects are per 1-SD increase in log_2-transformed values; brain-metastasis-count effects are per doubling. Direction agreement is the percentage of successful bootstrap fits in which the coefficient had the same sign as the corresponding original-model estimate.

B1. Additional record-abstraction batch adjustment

n = 91; DBF events = 57; competing deaths = 16.

Marker	Adjusted sHR (95% CI)	P value	q value
NLR	1.51 (1.09–2.10)	0.014	0.054
PLR	1.38 (1.04–1.83)	0.027	0.054
SII	1.37 (0.99–1.89)	0.057	0.076
LMR	0.80 (0.60–1.05)	0.113	0.113

B2. Additional GKRS-year adjustment.

n = 91; DBF events = 57; competing deaths = 16.

Marker	Adjusted sHR (95% CI)	P value	q value
NLR	1.52 (1.09–2.10)	0.012	0.033
PLR	1.43 (1.07–1.92)	0.017	0.033
SII	1.38 (1.00–1.90)	0.051	0.067
LMR	0.77 (0.58–1.03)	0.077	0.077

B3. Additional baseline-KPS adjustment

n = 91; DBF events = 57; competing deaths = 16.

Marker	Adjusted sHR (95% CI)	P value	q value
NLR	1.60 (1.18–2.18)	0.003	0.011

PLR	1.44 (1.09–1.89)	0.009	0.018
SII	1.47 (1.08–2.00)	0.014	0.018
LMR	0.74 (0.56–0.97)	0.030	0.030

B4. Minimum equivalent dose

n = 91; DBF events = 57; competing deaths = 16.

Marker	Adjusted sHR (95% CI)	P value	q value
NLR	1.54 (1.11–2.13)	0.009	0.037
PLR	1.40 (1.05–1.86)	0.020	0.040
SII	1.40 (1.01–1.95)	0.042	0.056
LMR	0.78 (0.59–1.04)	0.087	0.087

B5. Treated-lesion count

n = 91; DBF events = 57; competing deaths = 16.

Marker	Adjusted sHR (95% CI)	P value	q value
NLR	1.50 (1.09–2.07)	0.014	0.041
PLR	1.39 (1.05–1.84)	0.021	0.041
SII	1.38 (1.01–1.90)	0.046	0.061
LMR	0.78 (0.59–1.03)	0.074	0.074

B6. Exclusion of prior WBRT

n = 74; DBF events = 48; competing deaths = 12.

Marker	Adjusted sHR (95% CI)	P value	q value
NLR	1.69 (1.22–2.36)	0.002	0.007
PLR	1.34 (0.99–1.81)	0.054	0.097
SII	1.37 (0.97–1.92)	0.073	0.097
LMR	0.92 (0.69–1.23)	0.580	0.580

B7. Lower-burden subset

n = 86; DBF events = 52; competing deaths = 16.

Marker	Adjusted sHR (95% CI)	P value	q value
NLR	1.52 (1.10–2.10)	0.011	0.043
PLR	1.35 (0.99–1.83)	0.054	0.076

SII	1.36 (0.99–1.87)	0.057	0.076
LMR	0.80 (0.59–1.08)	0.150	0.150

B8. Endpoint-flag exclusion

n = 88; DBF events = 55; competing deaths = 16.

Marker	Adjusted sHR (95% CI)	P value	q value
NLR	1.57 (1.13–2.17)	0.007	0.028
PLR	1.40 (1.06–1.86)	0.019	0.039
SII	1.38 (0.99–1.91)	0.057	0.070
LMR	0.77 (0.58–1.02)	0.070	0.070

Notes: Each sensitivity analysis modified one aspect of the corresponding primary Fine–Gray model. R*ecord-abstraction batch* adjustment added the two-batch indicator, with the 69-record batch as reference; *bat*c*h* and calendar period were linked. GKRS year was centered on the full-cohort mean; baseline KPS was entered per 10 points. Minimum equivalent dose replaced volume-weighted equivalent dose, and $\log_2$ treated-lesion count replaced $\log_2$ total baseline brain-metastasis count. The prior-WBRT exclusion analysis omitted the resulting constant WBRT term. The lower-burden subset excluded patients with >10 baseline brain metastases or cumulative treated volume >25 cm^3. Endpoint-flag exclusion removed three patients whose records requested review of whether an intracranial event was outside the original treatment target (two DBF events and one censored observation). Original transformation parameters were retained. BH-FDR correction was applied separately to the four marker tests within each analysis. Figure S3C shows the ranges across 91 leave-one-patient-out estimates; these are not confidence intervals.

Table S4. Restricted cubic spline analyses of pretreatment NLR

Outcome and model	Patients, n	Events, n	Test	df	Wald χ^2	P value
First DBF (Fine–Gray)	91	57	NLR overall	2	5.72	0.057
First DBF (Fine–Gray)	91	57	NLR nonlinearity	1	1.08	0.298
Death after DBF (Cox)	57	36	NLR overall	2	16.83	<0.001
Death after DBF (Cox)	57	36	NLR nonlinearity	1	0.38	0.539

Notes: Splines used three knots at the full-cohort 10th, 50th, and 90th percentiles of $\log_2$-standardized NLR, with the cohort median as reference. First-DBF models included the primary clinical adjustment

set. Post-DBF models adjusted for age, KPS at DBF, progressive extracranial disease at DBF, and KPS × log(t/182.625). Overall and nonlinearity P values are nominal Wald tests with two and one degrees of freedom, respectively. See Figure S4.

Table S5. Time-varying inflammatory-marker associations with first distant brain failure

A. First DBF Fine–Gray time interaction tests

All models included 91 patients, 57 DBF events, 16 competing deaths, and nine coefficients.

Marker	Time coefficient γ	SE of γ	Interaction P	Interaction q	Overall P (2 df)
NLR	-0.125	0.095	0.189	0.694	<0.001
PLR	0.097	0.153	0.525	0.700	0.045
SII	-0.115	0.122	0.347	0.694	0.085
LMR	0.024	0.084	0.774	0.774	0.261

Notes: The time interaction was $\gamma z \log(t/365.25)$, where z is the standardized $\log_2$ marker and t is days since first GKRS. The marker main effect and all original clinical covariates were retained. Interaction P tests $\gamma = 0$; overall P tests $\beta = \gamma = 0$ jointly. The q values cover the four interaction tests only, not the overall tests or the original constant-effect models.

B. Time-specific first DBF associations

Marker	Months after GKRS	Adjusted sHR (95% CI)	Event-free at risk, n
NLR	6	1.49 (1.09–2.03)	62
NLR	12	1.36 (0.92–2.02)	44
NLR	24	1.25 (0.77–2.04)	12
PLR	6	1.38 (1.04–1.82)	62
PLR	12	1.47 (1.08–2.02)	44
PLR	24	1.58 (1.00–2.48)	12
SII	6	1.37 (1.00–1.88)	62
SII	12	1.26 (0.88–1.82)	44
SII	24	1.17 (0.73–1.87)	12
LMR	6	0.79 (0.60–1.05)	62
LMR	12	0.80 (0.60–1.07)	44
LMR	24	0.82 (0.58–1.15)	12

Notes: Marker effects are per 1-SD increase in log_2-transformed values. Models adjusted for baseline brain-metastasis count, age, molecular subtype, extracranial metastasis, prior WBRT, and volume-weighted equivalent single-fraction dose. Intervals are pointwise and use the complete covariance matrix of the main effect and interaction. Risk counts are ordinary event-free counts, not Fine–Gray weighted risk sets. CI, confidence interval; DBF, distant brain failure; df, degrees of freedom; GKRS, Gamma Knife radiosurgery; SD, standard deviation; SE, standard error; sHR, subdistribution hazard ratio; WBRT, whole-brain radiotherapy.

Table S6. Inflammatory-marker associations with mortality before and after distant brain failure

A. Inflammatory-marker associations with mortality

Transition	Marker	Patients, n	Deaths, n	Adjusted HR (95% CI)	P value	q value
Death before DBF	NLR	91	16	1.46 (0.78–2.73)	0.234	0.528
Death before DBF	PLR	91	16	0.75 (0.43–1.32)	0.322	0.528
Death before DBF	SII	91	16	1.16 (0.61–2.21)	0.654	0.745
Death before DBF	LMR	91	16	1.18 (0.55–2.53)	0.678	0.745
Death after DBF	NLR	57	36	2.29 (1.45–3.61)	<0.001	0.003
Death after DBF	PLR	57	36	1.07 (0.71–1.62)	0.745	0.745
Death after DBF	SII	57	36	1.32 (0.85–2.06)	0.210	0.528
Death after DBF	LMR	57	36	0.78 (0.47–1.29)	0.330	0.528

Notes: Each marker was entered into a separate Cox model, with effects per 1-SD increase in log_2-transformed values standardized using all 91 patients. Death-before-DBF models adjusted for age and baseline KPS; death-after-DBF models adjusted for age, KPS at DBF, and progressive extracranial disease at DBF. Models used Efron ties. Panel A q values represent BH-FDR correction across the eight marker tests spanning the two mortality transitions.

B. Proportional-hazards diagnostics for death-before-DBF models

n = 91; events = 16.

Marker model	Term or test	χ^2	df	Diagnostic P value
NLR	Pretreatment NLR, per 1-SD log_2 increase	1.82	1	0.177
NLR	Age, per 10 years	1.50	1	0.220
NLR	Baseline KPS, per 10 points	6.07	1	0.014

NLR	Global test	14.08	3	0.003
PLR	Global test	6.86	3	0.077
SII	Global test	10.44	3	0.015
LMR	Global test	5.12	3	0.163

C. Proportional-hazards diagnostics for death-after-DBF models

n = 57; events = 36.

Marker model	Term or test	χ^2	df	Diagnostic P value
NLR	Pretreatment NLR, per 1-SD $\log_2$ increase	2.47	1	0.116
NLR	Age, per 10 years	0.07	1	0.786
NLR	KPS at DBF, per 10 points	7.12	1	0.008
NLR	Progressive extracranial disease at DBF vs absent/stable	1.96	1	0.161
NLR	Global test	9.20	4	0.056
PLR	Global test	4.18	4	0.382
SII	Global test	4.97	4	0.291
LMR	Global test	4.32	4	0.365

Notes: Scaled Schoenfeld-residual tests used the Kaplan–Meier time transformation. Term-specific and global tests are shown for NLR models; global tests are also shown for parallel PLR, SII, and LMR models. These P values assess proportional hazards rather than covariate associations. Cox diagnostics do not assess the Fine–Gray proportional subdistribution-hazards assumption. DBF, distant brain failure; HR, hazard ratio; CI, confidence interval; SD, standard deviation; KPS, Karnofsky performance status; NLR, neutrophil-to-lymphocyte ratio; PLR, platelet-to-lymphocyte ratio; SII, systemic immune-inflammation index; LMR, lymphocyte-to-monocyte ratio; BH-FDR, Benjamini–Hochberg false discovery rate; df, degrees of freedom.

Table S7. Survival estimates after distant brain failure

Months after DBF	Survival, % (95% CI)	At risk, n
6	60.02 (45.83–71.61)	31
12	42.31 (28.38–55.57)	17

24	35.84 (21.80–50.10)	8

Notes: Median survival after DBF was 10.5 months (95% CI, 5.5–26.7), based on complete follow-up in 57 patients with 36 deaths. Survival after DBF used time reset at DBF. One month equals 365.25/12 days. Kaplan–Meier intervals use Greenwood variance and a log–log transformation. Risk counts refer to observed patients immediately before each time point. DBF, distant brain failure; CI, confidence interval.

Table S8. Additional analyses of mortality after distant brain failure

A1. Time-interaction coefficients after DBF

**Model**	**Interaction term**	**Parameters, n**	**γ**	**SE**	**P value**
KPS time-varying; NLR constant	KPS time interaction	5	−0.411	0.183	0.025
NLR time-varying; KPS constant	NLR time interaction	5	−0.322	0.225	0.153
NLR and KPS time-varying	NLR time interaction	6	−0.211	0.222	0.342
NLR and KPS time-varying	KPS time interaction	6	−0.375	0.190	0.048

A2. Joint test of NLR association

**Model**	**Test**	**df**	**Wald χ^2**	**P value**
NLR and KPS time-varying	NLR main effect and time interaction	2	16.00	<0.001

A3. NLR association with time-varying KPS adjustment

**Model**	**NLR HR (95% CI)**	**P value**
KPS time-varying; NLR constant	2.75 (1.68–4.51)	<0.001

A4. Time-specific effects in the model with both NLR and KPS time interactions

**Variable**	**Months after DBF**	**HR(t) (95% CI)**	**At risk, n**
Pretreatment NLR, per 1-SD $\log_2$ increase	3	2.88 (1.71–4.83)	43
Pretreatment NLR, per 1-SD $\log_2$ increase	6	2.48 (1.44–4.27)	31
Pretreatment NLR, per 1-SD $\log_2$ increase	12	2.15 (1.06–4.36)	17

KPS at DBF, per 10 points	3	0.57 (0.46–0.71)	43
KPS at DBF, per 10 points	6	0.44 (0.30–0.65)	31
KPS at DBF, per 10 points	12	0.34 (0.18–0.63)	17

Notes: All models included 57 patients with 36 deaths and adjusted for pretreatment NLR, age, KPS at DBF, and progressive extracranial disease at DBF. Time interactions used log(t/182.625), where t is time in days after DBF. NLR effects are per 1-SD increase in full-cohort $\log_2$-standardized values. For covariates with time interactions, main-effect HRs refer to six months after DBF. Time-specific effects were calculated as $HR(t) = \exp[\beta + \gamma \log(t/182.625)]$, with pointwise intervals incorporating covariance. γ is the interaction coefficient; exp(γ) is not a time-specific HR. P values are nominal. See Figure S6.

B1. Additional molecular-subtype adjustment after DBF

Model	Parameters, n	NLR HR (95% CI)	NLR P value	Δβ for NLR	Subtype P value
Constant NLR and KPS	6	2.21 (1.37–3.56)	0.001	−0.035	0.891
Constant NLR; time-varying KPS	7	2.71 (1.60–4.59)	<0.001	−0.016	0.935

B2. Main-effect estimates after subtype adjustment

Variable	Constant KPS HR (95% CI)	P value	Time-varying KPS HR (95% CI)	P value
Pretreatment NLR, per 1-SD $\log_2$ increase	2.21 (1.37–3.56)	0.001	2.71 (1.60–4.59)	<0.001
Age, per 10 years	0.81 (0.51–1.28)	0.358	0.91 (0.57–1.46)	0.709
KPS at DBF, per 10 points[a]	0.58 (0.47–0.73)	<0.001	0.41 (0.28–0.60)	<0.001
Progressive extracranial disease at DBF vs absent/stable	1.82 (0.70–4.72)	0.217	1.89 (0.71–5.00)	0.200
Hormone receptor-positive/HER2-negative vs HER2-positive	1.34 (0.40–4.50)	0.641	1.09 (0.31–3.82)	0.888
Triple-negative vs HER2-positive	1.13 (0.39–3.28)	0.817	1.22 (0.42–3.58)	0.717

B3. KPS time-interaction coefficient after subtype adjustment

Model	Interaction term	γ	SE	P value

Subtype-adjusted; time-varying KPS	KPS × log(t/182.625)	−0.411	0.184	0.026

Notes: Both subtype-adjusted models included 57 patients and 36 deaths. NLR coefficients were constant. Molecular subtype was entered as a three-level variable with HER2-positive disease as reference. Other covariates were age, KPS at DBF, and progressive extracranial disease at DBF. Δβ is the change in the NLR log-HR relative to the corresponding model without subtype adjustment. Subtype P values are two-degree-of-freedom Wald tests; all P values are nominal. [a] In the time-varying KPS model, the KPS main-effect HR applies at six months after DBF.

C. Mortality after DBF on the GKRS time scale

Delayed-entry Cox models: 57 patients, 36 deaths; four coefficients per model. Each marker was entered separately.

Marker	Adjusted HR (95% CI)	P value
NLR	2.16 (1.30–3.58)	0.003
PLR	1.10 (0.72–1.68)	0.656
SII	1.27 (0.82–1.96)	0.284
LMR	0.74 (0.45–1.23)	0.247

Notes: Time was measured from first GKRS, with delayed entry at DBF. Models adjusted for age, KPS at DBF, and progressive extracranial disease at DBF. Effects are per 1-SD increase in $\log_2$-transformed pretreatment marker values, standardized in all 91 patients. Patients contributed post-DBF risk time only. Models used Efron ties and model-based variance; all converged. P values are nominal and are not part of the eight-test mortality family for the primary clock-reset analyses. DBF, distant brain failure; GKRS, Gamma Knife radiosurgery; HR, hazard ratio; CI, confidence interval; KPS, Karnofsky performance status; SD, standard deviation.

D. Additional disease-burden and treatment-line adjustment

All models included a time-varying KPS coefficient and a constant NLR coefficient. Each extended model and its reference model were fitted to the same complete-case sample.

D1. Site-count same-patient reference (56 patients, 35 deaths)

Variable	HR (95% CI), unless specified	Nominal P
Pretreatment NLR, per 1 SD $\log_2$	2.71 (1.64–4.46)	<0.001

Age, per 10 years	0.92 (0.61–1.39)	0.694
KPS at DBF, per 10 points (6-month effect)	0.43 (0.29–0.62)	<0.001
Extracranial progression, yes vs no	1.92 (0.78–4.74)	0.154
KPS × log(time/182.625), coefficient γ (SE)	-0.387 (0.184)	0.036

D2. Additional site-count adjustment (56 patients, 35 deaths)

Variable	HR (95% CI), unless specified	Nominal P
Pretreatment NLR, per 1 SD $\log_2$	2.65 (1.61–4.38)	<0.001
Age, per 10 years	0.93 (0.62–1.38)	0.718
KPS at DBF, per 10 points (6-month effect)	0.41 (0.27–0.60)	<0.001
Extracranial progression, yes vs no	1.48 (0.56–3.95)	0.429
Metastatic sites, per additional site	1.42 (0.87–2.29)	0.157
KPS × log(time/182.625), coefficient γ (SE)	-0.433 (0.194)	0.025

D3. Treatment-line same-patient reference (54 patients, 33 deaths)

Variable	HR (95% CI), unless specified	Nominal P
Pretreatment NLR, per 1 SD $\log_2$	3.24 (1.87–5.61)	<0.001
Age, per 10 years	0.93 (0.61–1.43)	0.754
KPS at DBF, per 10 points (6-month effect)	0.39 (0.26–0.57)	<0.001
Extracranial progression, yes vs no	1.98 (0.78–5.02)	0.150
KPS × log(time/182.625), coefficient γ (SE)	-0.529 (0.210)	0.012

D4. Additional treatment-line adjustment (54 patients, 33 deaths)

Variable	HR (95% CI), unless specified	Nominal P
Pretreatment NLR, per 1 SD $\log_2$	3.13 (1.77–5.53)	<0.001
Age, per 10 years	0.94 (0.61–1.43)	0.760
KPS at DBF, per 10 points (6-month effect)	0.39 (0.26–0.59)	<0.001
Extracranial progression, yes vs no	1.88 (0.72–4.95)	0.199
Treatment line, per additional line	1.11 (0.74–1.67)	0.615
KPS × log(time/182.625), coefficient γ (SE)	-0.514 (0.213)	0.016

Notes: Reference and extended models contained five and six coefficients, respectively. NLR uses the

full-cohort $\log_2$ mean and SD. The KPS main effect applies at six months; its interaction is reported as γ (SE), not as a time-specific HR. One site count and three treatment-line values were missing. CI, confidence interval; DBF, distant brain failure; HR, hazard ratio; KPS, Karnofsky performance status; NLR, neutrophil-to-lymphocyte ratio; SD, standard deviation; SE, standard error.

Table S9. Treatment timing and time-dependent associations with mortality after distant brain failure

A. Treatment timing and observation summaries

Exposure	Delay, days, median (IQR)	Start date recorded, n/N	Person-days before / after initiation	Deaths before / after initiation
Salvage GKRS	26.5 (17.8–50.0)	34/34	6967 / 12368	17 / 19
Systemic treatment initiation/change	10.5 (5.0–17.5)	44/44	3896 / 15439	11 / 25

B. Treatment initiation and adjusted mortality associations

Time-dependent exposure	Initiated, n/N	Not initiated, n	Adjusted HR (95% CI)	P value	q value
Salvage GKRS	34/57	23	0.86 (0.40–1.85)	0.702	0.702
Systemic treatment initiation/change	44/57	13	0.19 (0.07–0.50)	<0.001	0.001

Notes: Both analyses included 57 patients with 36 deaths, with follow-up beginning at first DBF. Each treatment was evaluated separately in a six-coefficient Cox model with Efron ties and model-based variance. Models included the treatment indicator, pretreatment $\log_2$-standardized NLR, age per 10 years, KPS at DBF per 10 points, progressive extracranial disease at DBF, and KPS $\times$ $\log(t/182.625)$. Exposure switched from 0 before the documented start to 1 after initiation; patients initiating on the DBF date were exposed from the start of follow-up. Exposure represented initiation rather than continued treatment. BH-FDR correction covered the two treatment coefficients. Delay was summarized among initiators. Person-time and death counts are descriptive. The two models did not mutually adjust for the other treatment; estimates represent associations rather than causal treatment effects.

Table S10. Screening, surveillance, treatment characteristics, and model complexity

A. Recorded exclusion reasons

Exclusion reason (overlap allowed)	Patients, n
Corticosteroid exposure	16
Hematopoietic growth-factor or related drug exposure	19
Anti-inflammatory drug exposure	5
Hematologic disease	4
Autoimmune disease	3
Fever	3
Insufficient blood-count information	5
Insufficient follow-up information	10

Notes: The institutional screening register comprised 133 study candidates, with 42 excluded and 91 included. Exclusion reasons could overlap, so their total exceeded the number of excluded patients. All 16 corticosteroid-associated exclusions had recorded exposure dates within 1–14 days before GKRS.

B. Blood-sampling and MRI intervals

Measure	Available n	Median (IQR), days	Range, days
Blood sampling to GKRS	91	1 (1–1)	0–6
First recorded post-GKRS MRI	86 patients	77.5 (47.5–117.3)	17–344
Consecutive MRI intervals, pooled	210 intervals	98.0 (68.5–138.8)	12–378
Within-patient median MRI interval	72 patients	101.8 (77.0–141.8)	12–284
Preceding recorded MRI to first-DBF MRI	44 patients	102.5 (64.0–189.8)	12–284

Notes: MRI summaries include 296 distinct examination dates within the first-event observation windows of 86 patients, including confirmed first-DBF MRI dates. Five patients had no available post-GKRS MRI dates within this observation window; absence of a recorded date does not establish that no examination was performed. The preceding MRI-to-DBF interval does not establish that the preceding

scan was negative. Seventeen blood samples were collected on the GKRS date and confirmed to precede irradiation. Recommended MRI timing was 3 and 6 months, then every 6 months through year 3, with earlier assessment for relevant symptoms. Actual timing varied. IQR, interquartile range; MRI, magnetic resonance imaging.

C. Systemic treatment and extracranial burden information

Information	Patients / total	Summary
Specific current regimen at first GKRS	78/91	85.7%
No current regimen recorded at first GKRS	10/91	11.0%
Current regimen unknown at first GKRS	3/91	3.3%
HER2 TKI-containing regimen at first GKRS	28/91	30.8%
HER2 ADC-containing regimen at first GKRS	18/91	19.8%
Trastuzumab deruxtecan-containing regimen at first GKRS	12/91	13.2%
Trastuzumab emtansine-containing regimen at first GKRS	5/91	5.5%
Capecitabine-containing regimen at first GKRS	36/91	39.6%
Endocrine-containing regimen at first GKRS	5/91	5.5%
Other recorded regimens without a HER2 TKI or ADC at first GKRS	35/91	38.5%; may include the capecitabine or endocrine categories above
Extracranial metastatic-site count at DBF	56/57	Median 2 (IQR, 1–2); range 0–3
Recorded treatment line at DBF	54/57	Median 2 (IQR, 1–3); range 1–6
Post-DBF systemic initiation/change	44/57	77.2%; actual post-DBF initiation/change dates

Notes: First-GKRS treatment percentages use all 91 patients as the denominator. Categories reflect the

specific regimen recorded at first GKRS and may overlap; three patients had both a HER2-TKI and a HER2-ADC recorded. The 18 HER2-ADC regimens comprised trastuzumab deruxtecan (n = 12), trastuzumab emtansine (n = 5), and RC48 (n = 1), with no overlap between these ADC subcategories. Ten patients had no current regimen recorded and three had an unknown regimen. These categories are distinct from previous exposure and from systemic treatment initiated or changed after DBF. DBF treatment lines use the values recorded for that time point; uniform line-transition rules and dated source documentation were not available for every entry. DBF, distant brain failure; GKRS, Gamma Knife radiosurgery; HER2, human epidermal growth factor receptor 2; ADC, antibody–drug conjugate; TKI, tyrosine kinase inhibitor; IQR, interquartile range.

D. Model complexity

Model class	Patients / events	Coefficients	Events per coefficient
First-DBF primary marker models	91 / 57	8	7.13
First-DBF KPS/batch/year extension, spline or time interaction	91 / 57	9	6.33
Death before DBF	91 / 16	3	5.33
Death after DBF, constant coefficients	57 / 36	4	9.00
Death after DBF, KPS or NLR time interaction	57 / 36	5	7.20
Death after DBF, both time interactions or NLR spline	57 / 36	6	6.00
Post-DBF subtype adjustment, constant / time-varying KPS	57 / 36	6 / 7	6.00 / 5.14
Time-dependent treatment models	57 / 36	6	6.00
Additional metastatic-site count	56 / 35	6	5.83
Additional treatment line	54 / 33	6	5.50
First-DBF NLR only / GPA only / NLR plus GPA	90 / 56	1 / 1 / 2	56.00 / 56.00 / 28.00

Notes: Counts include two coefficients for three-category subtype and separate coefficients for spline and time-interaction terms. Events per coefficient are reported as descriptive measures of model complexity. Nonparametric CIF and Aalen–Johansen estimates do not use these regression adjustment

coefficients. Definitions and sample sizes for the eight first-DBF sensitivity analyses are provided in Table S3. DBF, distant brain failure; GKRS, Gamma Knife radiosurgery; KPS, Karnofsky performance status; NLR, neutrophil-to-lymphocyte ratio; GPA, graded prognostic assessment; CIF, cumulative incidence function.

Table S11. Updated Breast-GPA supplementary association analysis

Model	Variable	sHR (95% CI)	Nominal P
NLR only	NLR, per 1 SD $\log_2$	1.75 (1.23–2.48)	0.002
GPA only	GPA, per point	0.89 (0.64–1.25)	0.498
NLR plus GPA	NLR, per 1 SD $\log_2$	1.74 (1.23–2.46)	0.002
NLR plus GPA	GPA, per point	0.98 (0.66–1.44)	0.901

Notes: All three Fine–Gray models included the same 90 patients with available baseline updated Breast-GPA scores: 56 first-DBF events, 16 competing deaths, and 18 censored observations. Follow-up began at first GKRS. NLR effects are per 1-SD increase in $\log_2$ NLR, using the mean and sample SD of the full 91-patient cohort; GPA effects are per one-point increase. The joint model included only NLR and GPA, without separate GPA components. The missing GPA score was not imputed. P values are nominal and do not belong to the primary four-marker FDR family. GPA, graded prognostic assessment; NLR, neutrophil-to-lymphocyte ratio; SD, standard deviation; sHR, subdistribution hazard ratio; CI, confidence interval; DBF, distant brain failure; GKRS, Gamma Knife radiosurgery; FDR, false discovery rate.

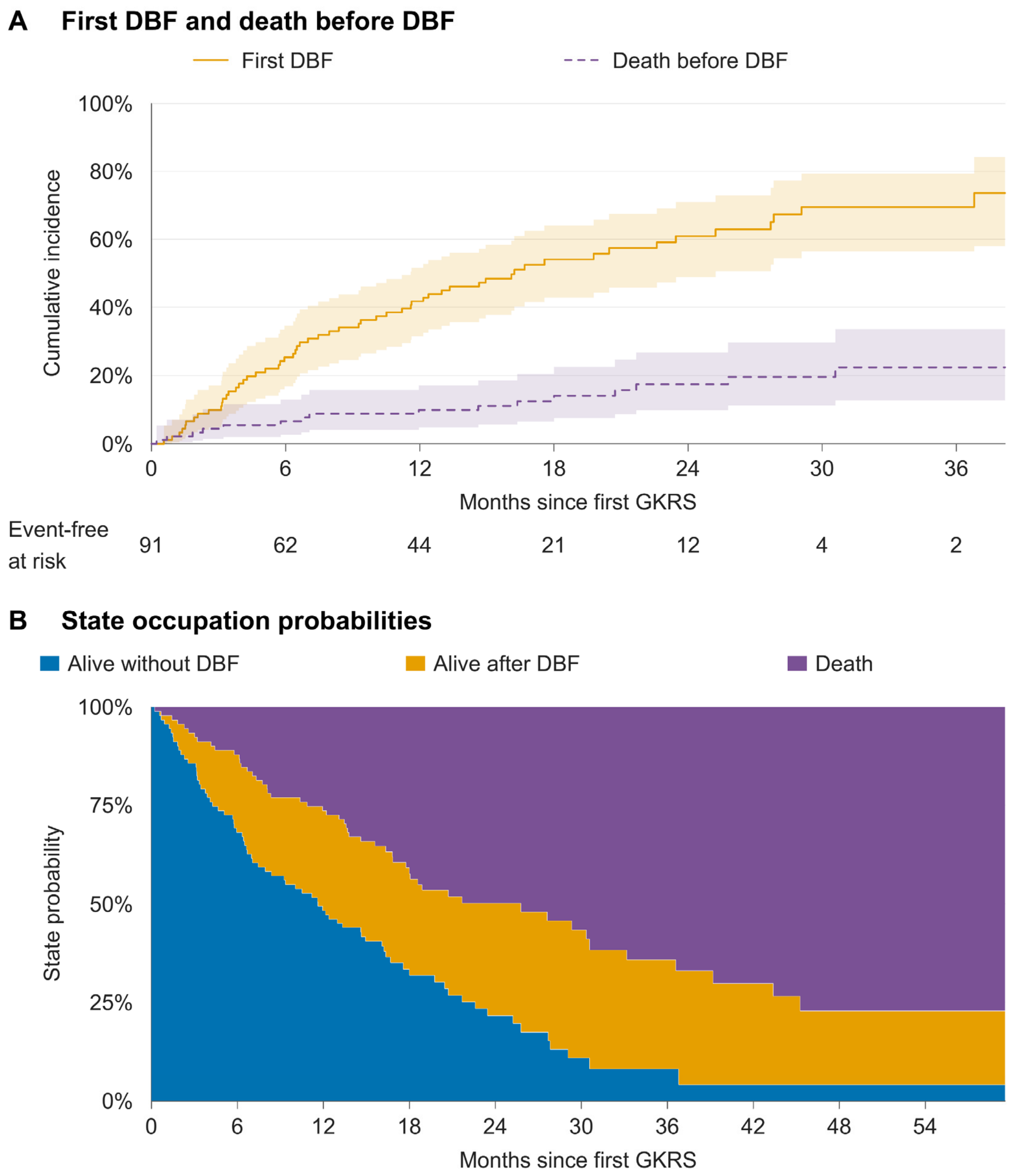

Figure S1. Cumulative incidence and disease-state probabilities over complete follow-up.

(A) Cumulative incidence of first distant brain failure (DBF; orange) and death before DBF (purple) in 91 patients, with pointwise 95% confidence intervals calculated using Aalen variance and a log–log transformation. The risk table shows patients still observed without either first event immediately before each time point. (B) Aalen–Johansen estimates of the probabilities of being alive without DBF (blue), alive after DBF (orange), or dead (purple). Curves show point estimates, and death includes both mortality transitions. The probability of being alive after DBF is distinct from the cumulative incidence of DBF. Time is measured from first Gamma Knife radiosurgery (GKRS). The follow-up

cutoff date was 1 July 2026. Each panel displays the complete follow-up range included in the analysis through that cutoff; Figure 2 shows the first 24 months of the same analyses. Complete survival after DBF is shown in Figure S5. One month equals 365.25/12 days.

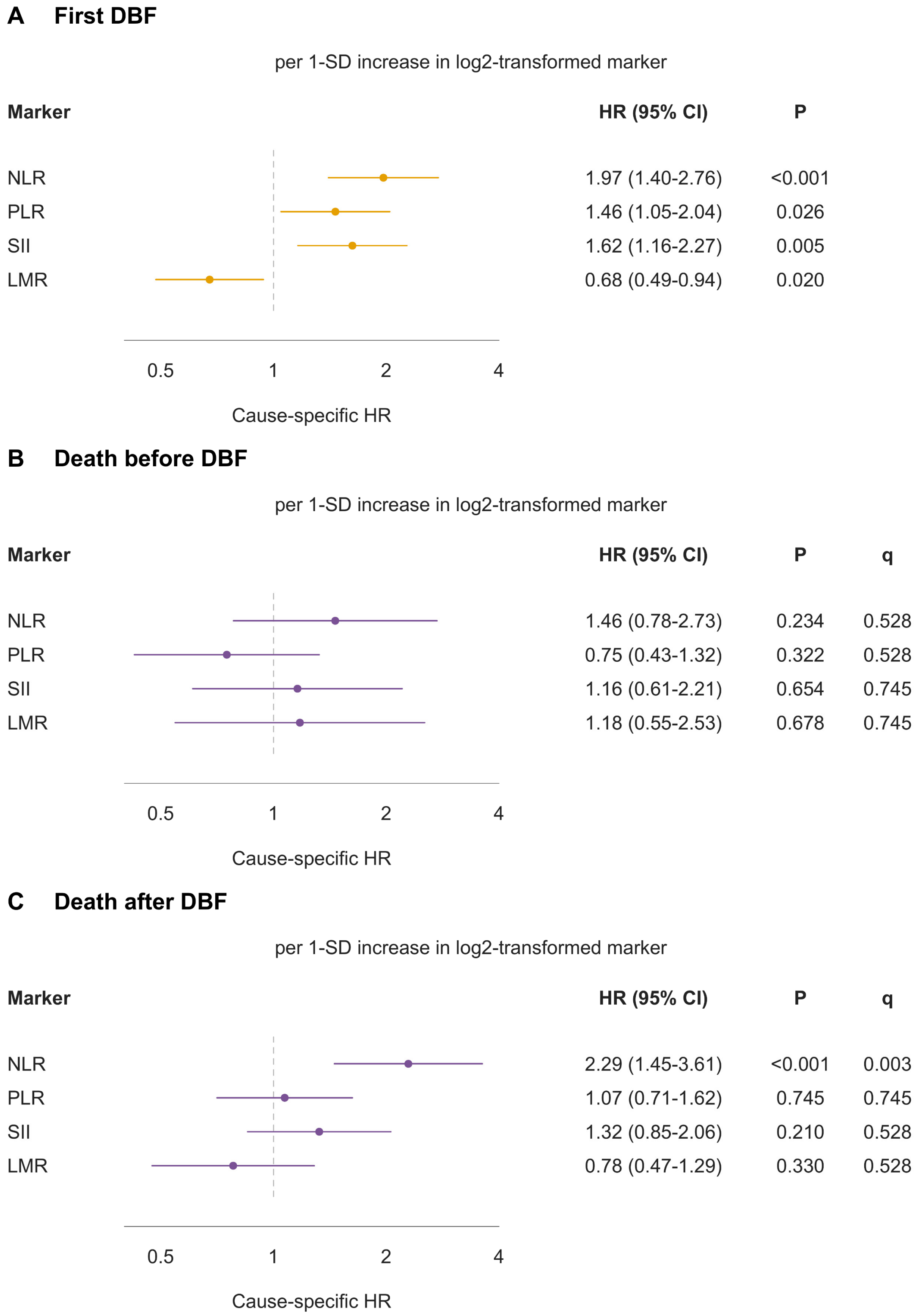

Figure S2. Inflammatory-marker associations across three disease transitions.

Cause-specific Cox hazard ratios (HRs) with 95% confidence intervals for (A) first distant brain failure (DBF), (B) death before DBF, and (C) death after DBF. Each marker was entered into a separate model, with effects per 1-SD increase in log_2-transformed values standardized using all 91 patients. In A,

follow-up began at first Gamma Knife radiosurgery (GKRS; 91 patients, 57 DBF events); death ended the risk interval. Models adjusted for baseline brain-metastasis count, age, molecular subtype, extracranial metastases, prior WBRT, and volume-weighted equivalent single dose. In B, follow-up began at GKRS (91 patients, 16 deaths); DBF ended the risk interval. Models adjusted for age and baseline KPS. In C, time was reset at DBF (57 patients, 36 deaths), with adjustment for age, KPS at DBF, and progressive extracranial disease at DBF. Models used Efron's method for ties. The q values in B and C were calculated across the eight adjusted marker tests as one BH-FDR family; panel A reports nominal P values. No formal comparison of effects across transitions was performed. DBF, distant brain failure; GKRS, Gamma Knife radiosurgery; KPS, Karnofsky performance status; NLR, neutrophil-to-lymphocyte ratio; PLR, platelet-to-lymphocyte ratio; SII, systemic immune-inflammation index; LMR, lymphocyte-to-monocyte ratio; CI, confidence interval; SD, standard deviation; HR, hazard ratio; sHR, subdistribution hazard ratio; WBRT, whole-brain radiotherapy; HER2, human epidermal growth factor receptor 2; BH-FDR, Benjamini–Hochberg false discovery rate.

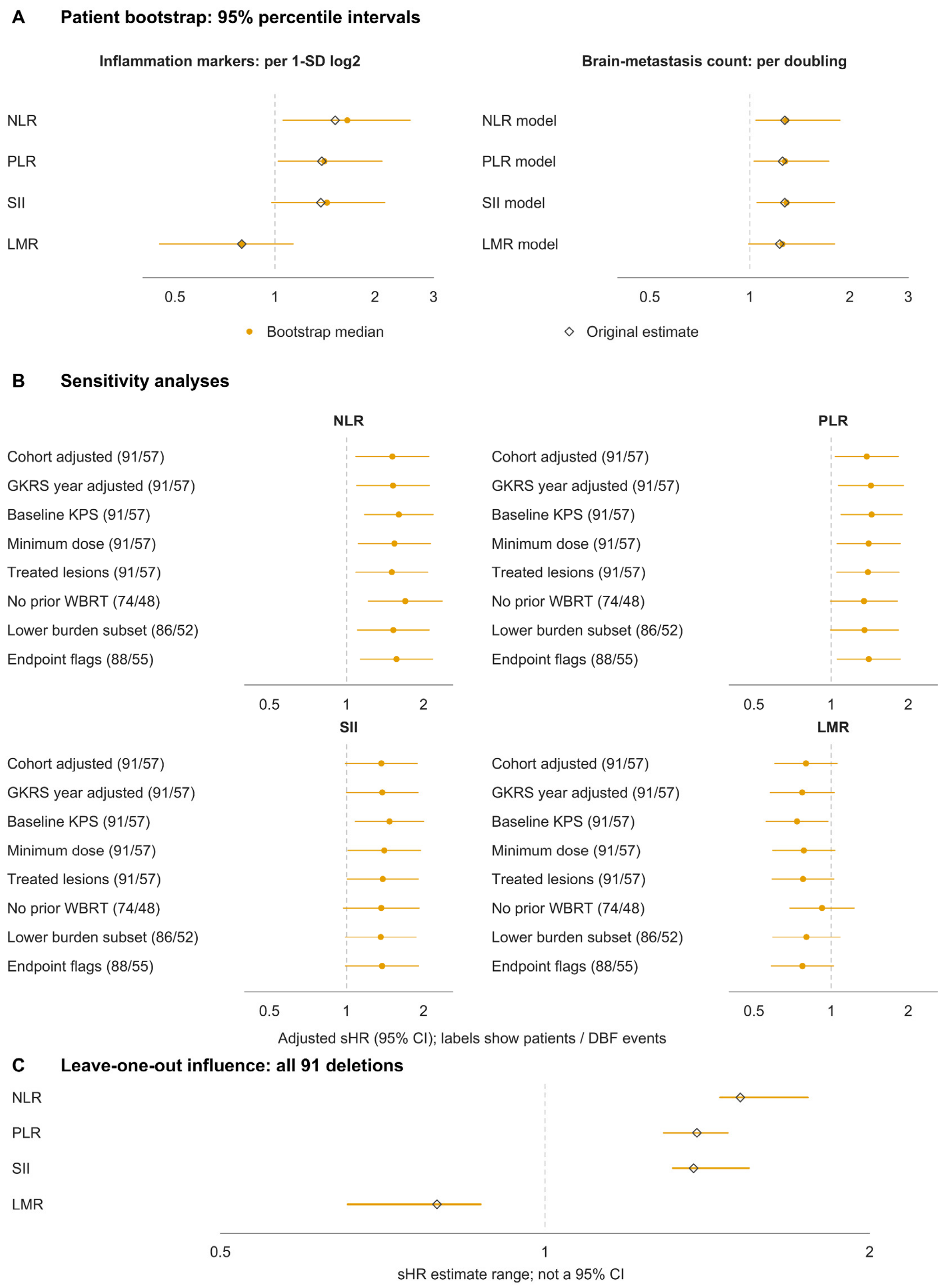

Figure S3. Bootstrap stability, sensitivity analyses, and influence diagnostics. (A) Median Fine–Gray subdistribution hazard ratios (sHRs; filled points) and 95% percentile intervals from 1,000 patient-level bootstrap resamples of 91 patients. Open diamonds indicate original estimates. The same resamples were used for all four marker models, retaining the original transformations and adjustment sets. Marker effects are per 1-SD increase in log_2-transformed values; brain-metastasis-count effects are per doubling. (B) Adjusted sHRs and 95% confidence intervals from

eight sensitivity analyses: additional adjustment for record-abstraction batch (the figure**'s cohort label denotes the two same-institution collection batches**), GKRS year, or baseline KPS; substitution of minimum equivalent dose for volume-weighted dose; substitution of treated-lesion count for total baseline count; exclusion of prior WBRT; restriction to ≤10 baseline brain metastases and cumulative treated volume ≤25 cm^3; and exclusion of patients carrying the recorded endpoint-adjudication flag. Labels show patient and DBF-event counts. Definitions and within-analysis four-marker BH-FDR q values are provided in (Table S3). (C) Ranges across 91 leave-one-patient-out sHR estimates, with original estimates shown as open diamonds. These ranges are not confidence intervals. DBF, distant brain failure; GKRS, Gamma Knife radiosurgery; KPS, Karnofsky performance status; NLR, neutrophil-to-lymphocyte ratio; PLR, platelet-to-lymphocyte ratio; SII, systemic immune-inflammation index; LMR, lymphocyte-to-monocyte ratio; CI, confidence interval; SD, standard deviation; HR, hazard ratio; sHR, subdistribution hazard ratio; WBRT, whole-brain radiotherapy; HER2, human epidermal growth factor receptor 2; BH-FDR, Benjamini–Hochberg false discovery rate.

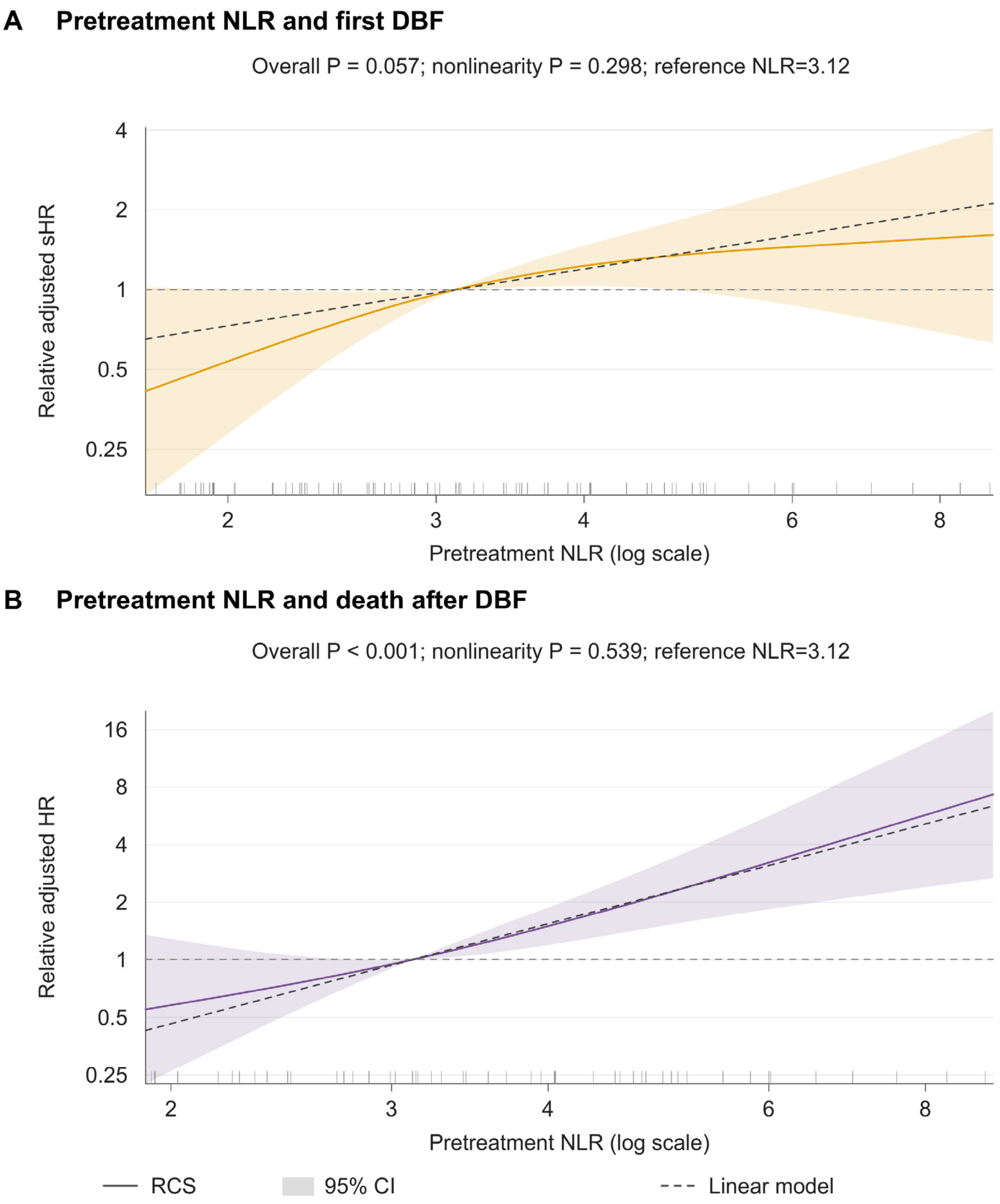

Figure S4. Shape of the association between pretreatment NLR and disease outcomes. (A) Relative subdistribution hazard for first distant brain failure (DBF) from a Fine–Gray model in 91 patients, adjusted for baseline brain-metastasis count, age, molecular subtype, extracranial metastases, prior WBRT, and volume-weighted equivalent single dose. (B) Relative hazard of death after DBF from a Cox model in 57 patients, adjusted for age, KPS at DBF, progressive extracranial disease at DBF, and KPS × log(t/182.625), where t is time since DBF in days. Restricted cubic splines used knots at the full-cohort 10th, 50th, and 90th percentiles of $\log_2$-standardized NLR, corresponding to raw NLR values of approximately 1.88, 3.12, and 6.54. The reference was the cohort median NLR of 3.12. Solid

lines show spline estimates, shading indicates pointwise 95% confidence intervals, and dashed lines show corresponding linear models. Rugs indicate observations within the display range; all eligible patients were included in model fitting. Overall and nonlinearity P values are nominal Wald tests with two and one degrees of freedom, respectively **(Table S4)**. NLR, neutrophil-to-lymphocyte ratio; KPS, Karnofsky performance status; WBRT, whole-brain radiotherapy.

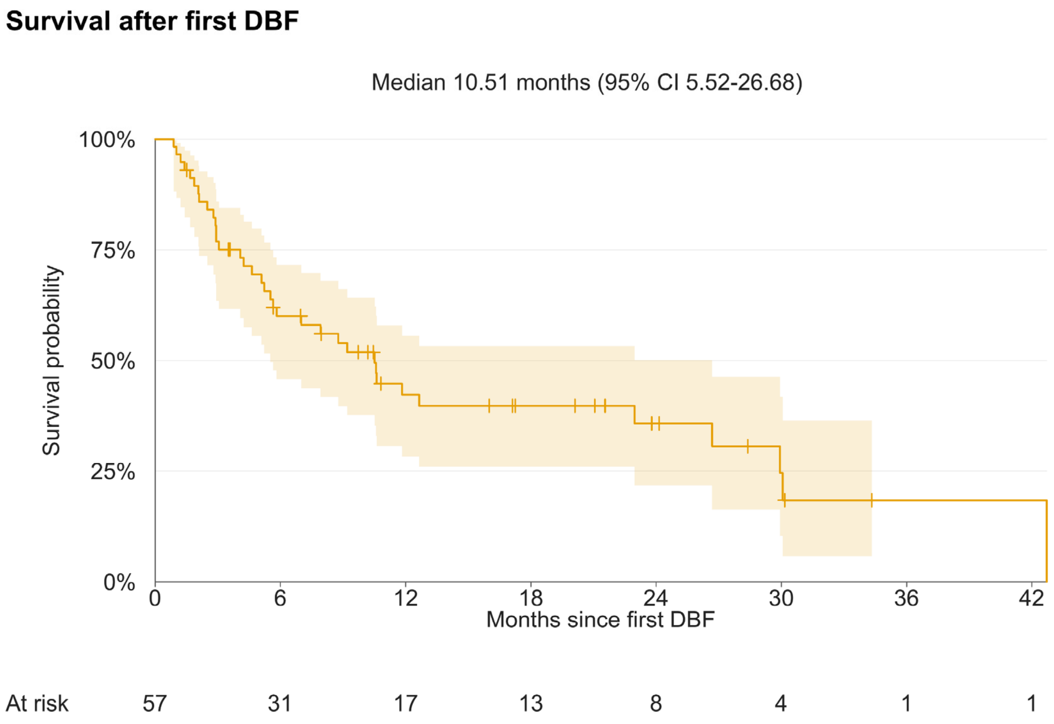

Figure S5. Survival after DBF over complete follow-up. Kaplan–Meier survival from first distant brain failure (DBF) in 57 patients, with 36 deaths. Shading indicates pointwise 95% confidence intervals calculated using Greenwood variance and a log–log transformation; plus signs indicate censoring. The risk table shows patients still under observation immediately before each time point. The median and its 95% confidence interval were estimated using complete follow-up. Figure 4A shows the first 24 months of the same survival estimate. One month equals 365.25/12 days.

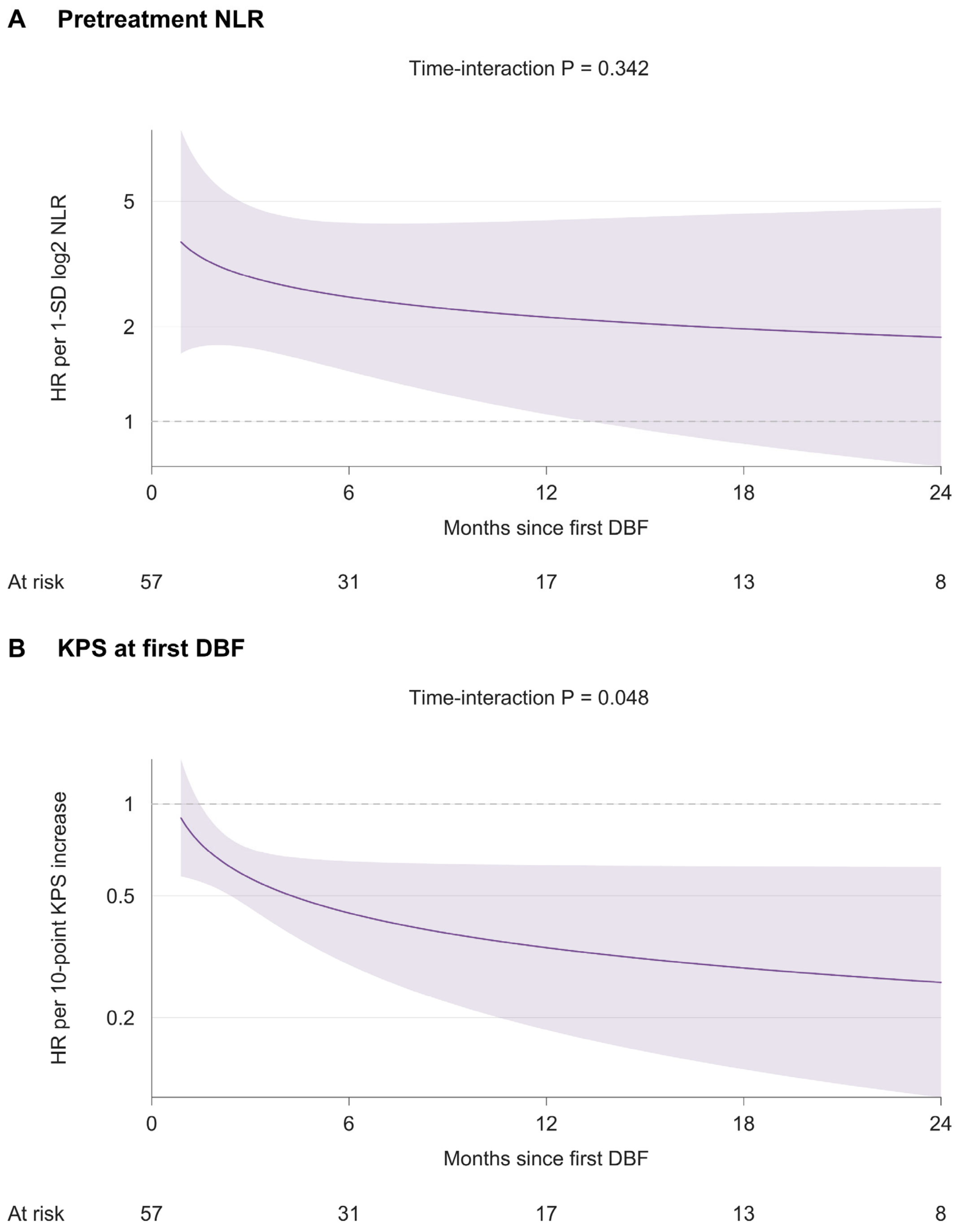

Figure S6. Time-varying associations of NLR and KPS with mortality after DBF.

(A) Pretreatment neutrophil-to-lymphocyte ratio (NLR), per 1-SD increase in full-cohort $\log_2$-standardized values. (B) Karnofsky performance status (KPS) at distant brain failure (DBF), per 10-point increase. The Cox model included NLR and KPS main effects and their interactions with log(t/182.625), with adjustment for age and progressive extracranial disease at DBF. Time t is measured in days after DBF, so main effects correspond to six months. Curves show HR(t) = exp[β + γ

log(t/182.625)], with pointwise 95% confidence intervals incorporating main-effect/interaction covariance. Displayed P values test the respective time interactions. Curves begin at the first observed positive death time; aligned risk tables describe the 57-patient cohort. NLR and KPS were fixed measurements, with coefficients allowed to vary over time. Numerical estimates are reported in **(Table S8A)**. One month equals 365.25/12 days. HR, hazard ratio; SD, standard deviation.

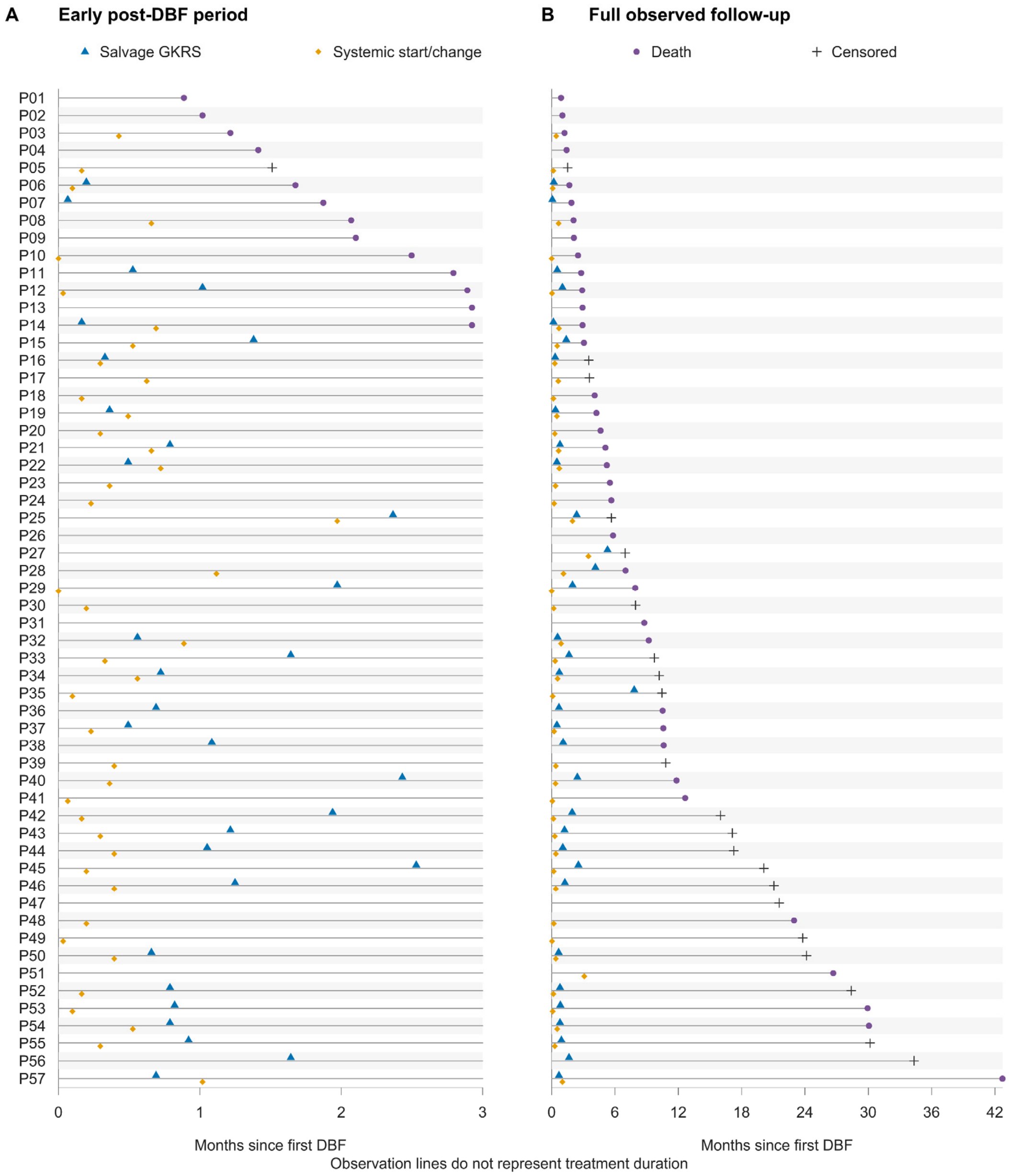

Figure S7. Individual treatment and outcome timelines after DBF.

(A) The first three months after distant brain failure (DBF). (B) Complete observed follow-up. Both panels show the same 57 patients in the same order, sorted by observed post-DBF duration. Blue triangles indicate salvage Gamma Knife radiosurgery; orange diamonds, systemic treatment initiation or change; purple circles, death; and plus signs, censoring. Each row represents one patient's observed follow-up, with symbols marking documented treatment and outcome dates. Panel A displays the first three months; panel B displays complete follow-up, including 36 deaths and 21 censored observations. Absence of a treatment marker does not imply absence of all treatment. One month equals 365.25/12 days.

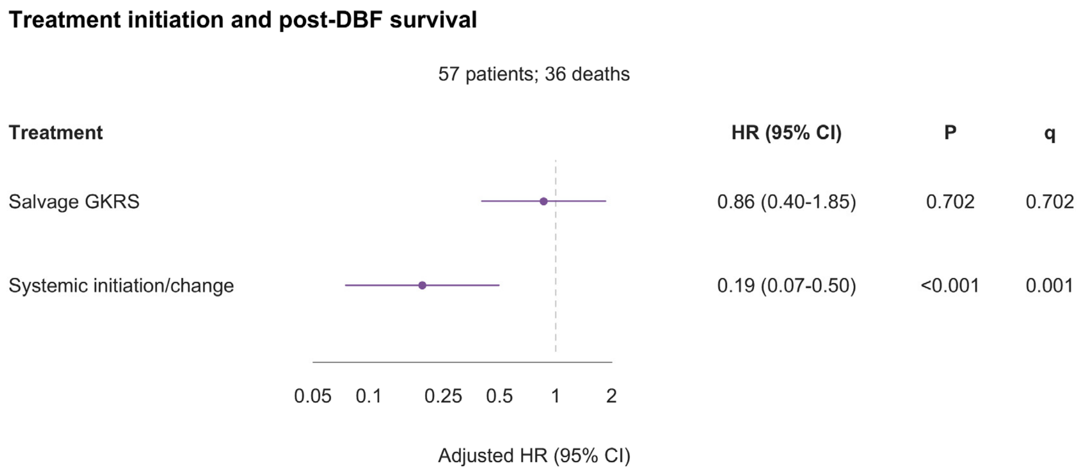

Figure S8. Time-dependent treatment initiation and survival after DBF.

Adjusted hazard ratios (HRs) and 95% confidence intervals for salvage Gamma Knife radiosurgery and systemic treatment initiation or change after distant brain failure (DBF; 57 patients, 36 deaths). Each treatment was evaluated in a separate Cox model as an exposure switching from 0 before its documented start to 1 after initiation. Models adjusted for pretreatment $\log_2$-standardized NLR, age, KPS at DBF, progressive extracranial disease at DBF, and KPS × log(t/182.625), where t is time since DBF in days. Models used Efron's method for ties and model-based variance estimates. The q values were calculated across the two treatment coefficients as one BH-FDR family. Exposure indicates initiation rather than continued treatment; absence of the specified initiation or change does not imply no previous or ongoing therapy. Estimates describe adjusted associations and do not establish causal treatment effects. NLR, neutrophil-to-lymphocyte ratio; KPS, Karnofsky performance status; BH-FDR, Benjamini–Hochberg false discovery rate.
